# The histone methyltransferase DOT1L inhibits osteoclastogenesis and protects against osteoporosis

**DOI:** 10.1038/s41419-017-0040-5

**Published:** 2018-01-18

**Authors:** Yanpan Gao, Wei Ge

**Affiliations:** 1 State Key Laboratory of Medical Molecular Biology & Department of Biochemistry and Molecular Biology, Institute of Basic Medical Sciences, Chinese Academy of Medical Sciences; School of Basic Medicine, Peking Union Medical College, Beijing, China; 2 State Key Laboratory of Medical Molecular Biology & Department of Immunology, Institute of Basic Medical Sciences, Chinese Academy of Medical Sciences; School of Basic Medicine, Peking Union Medical College, Beijing, China

## Abstract

Osteoclasts are absorptive cells that play a critical role in homeostatic bone remodeling and pathological bone resorption. Emerging evidence suggests an important role of epigenetic regulation in osteoclastogenesis. In this study, we investigated the role of DOT1L, which regulates gene expression epigenetically by histone H3K79 methylation (H3K79me), during osteoclast formation. Using RANKL-induced RAW264.7 macrophage cells as an osteoclast differentiation model, we found that DOT1L and H3K79me2 levels were upregulated during osteoclast differentiation. Small molecule inhibitor- (EPZ5676 or EPZ004777) or short hairpin RNA-mediated reduction in DOT1L expression promoted osteoclast differentiation and resorption. In addition, DOT1L inhibition increased osteoclast surface area and accelerated bone-mass reduction in a mouse ovariectomy (OVX) model of osteoporosis without alter osteoblast differentiation. DOT1L inhibition increase reactive oxygen species (ROS) generation and autophagy activity, and cell migration in pre-osteoclasts. Moreover, it strengthened expression of osteoclast fusion and resorption-related protein CD9 and MMP9 in osteoclasts derived from RAW264.7. Our findings support a new mechanism of DOT1L-regulated, H3K79me2-mediated, epigenetic regulation of osteoclast differentiation, implicating DOT1L as a new therapeutic target for osteoclast dysregulation-induced disease.

## Introduction

Osteoclasts (OCs) are the primary effectors of bone resorption and are indispensable for bone remodeling and repair and maintaining mineral homeostasis. Dysregulation of these large, multinucleate cells is the main cause of most bone disorders (nearly 90%) such as osteoporosis (OP) (systemic) or osteolysis (local), which are usually accompanied by spontaneous fractures and hypercalcemia^1^. Therefore, targeting and managing the formation and function of OCs might be effective for the prevention and treatment of bone disorders.

OC formation is triggered by a series of RANKL-induced signaling events that lead to activation of transcription factors such as NFATc1 and NF-κB^2,3^. These transcription factors induce the expression of OC-specific genes such as *TRAP*, *CTSK*, and *MMP9*. There are three stages of OC differentiation: pro-osteoclast (spindle-shaped macrophage cells), pre-osteoclast (pre-OC, small, round, mononucleated TRAP-positive cells), and mature osteoclast (OC, multinucleated TRAP-positive cells) stage^4^. Although RANKL-induced signaling pathways have been intensively studied in OC differentiation^5^, the roles of epigenetic regulation and histone methylation in regulation of the RANKL-induced pathway are not well elucidated. A previous study reported that downregulation of Jmjd3, which is an H3K27 demethylase, suppresses RANKL-induced osteoclastogenesis^6^. After RANKL stimulation, the H3K27 methyltransferase EZH2 downregulates the expression of IRF8, which is a negative regulator of osteoclastogenesis. Inhibition or downregulation of EZH2 impedes OC differentiation^7^. A specific inhibitor of H3K9 methyltransferase, G9a, is known to reduce expression of RANKL-induced, TRAP-positive OCs number^8^. NO66, which harbors histone demethylase activity, demethylates H3K4 and H3K36. *NO66*-knockout mice exhibit increased levels of H3K4 and H3K36 trimethylation (H3K4me3 and H3K36me3, respectively) and reduced OCs number, with low TRAP expression^9^. These findings indicate the importance of histone-methylation regulators in RANKL-induced osteoclastogenesis.

The H3K79 methyltransferase DOT1L is another important histone-methylation regulator. DOT1L-dependent H3K79 methylation plays a role in several processes such as embryonic development, erythropoiesis, mixed-lineage leukemia gene (MLL)-rearranged leukemia, cardiac differentiation^10^, chondrocyte differentiation^11^, and cartilage homeostasis^12^. However, the roles of DOT1L in OC regulation and OC-related disease have not been reported thus far.

Therefore, this study aimed to elucidate (1) the role of DOT1L in OC differentiation *in vitro* and (2) the effect of DOT1L on balance of trabecular bone metabolism *in vivo*.

## Results

### Knockdown of DOT1L enhances cell fusion and resorption activity in RAW264.7 osteoclastogenesis model

**Fig. 1:**
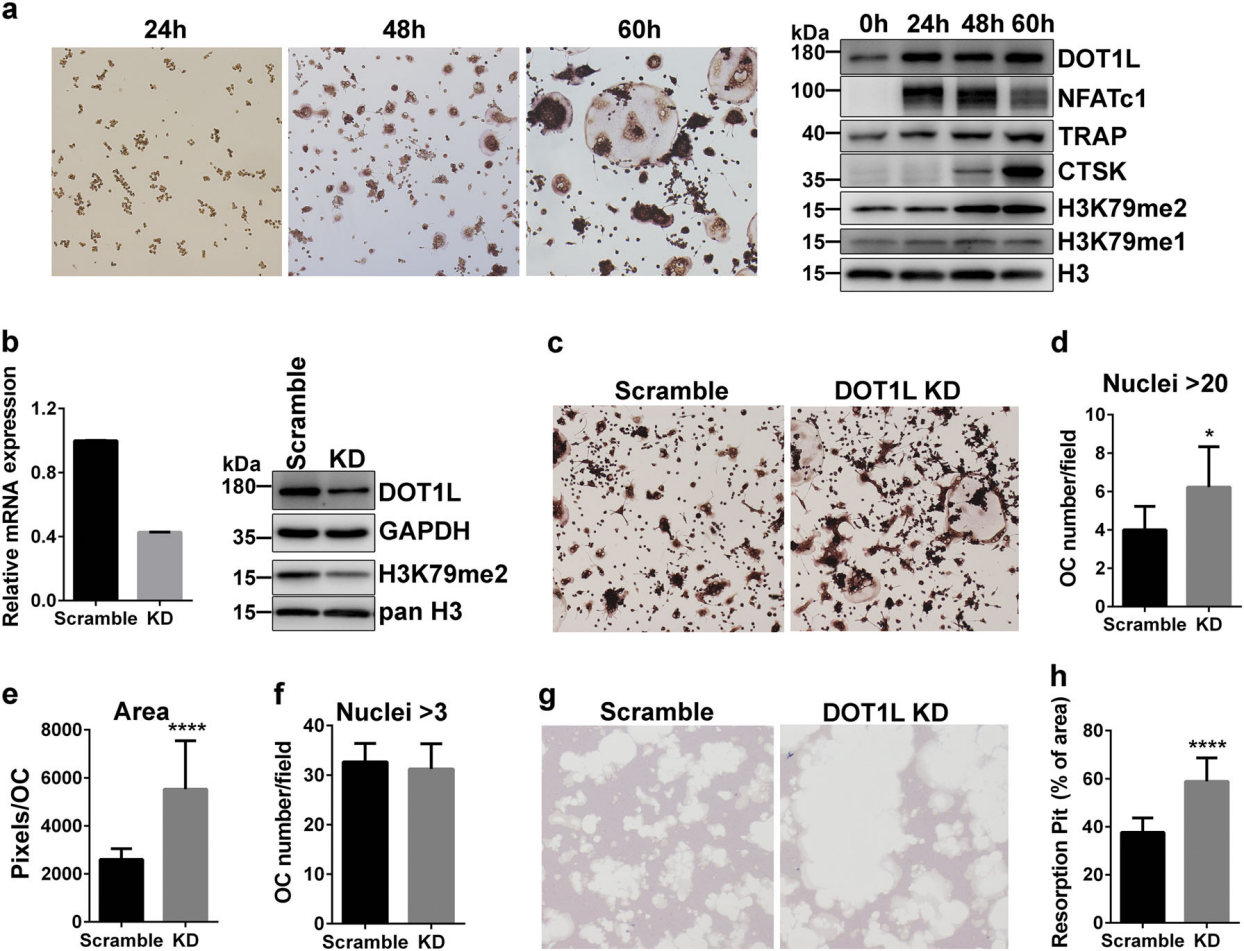
Roles of DOT1L in OC fusion and resorption **a** Murine RAW264.7 cells stimulated with 10 ng/mL RANKL for the indicated time. TRAP staining (left panel) was performed and protein expression (right panel) of DOT1L, NFATc1, TRAP, CTSK, H3K79me1, and H3K79me2 in response to RANKL stimulation was detected by western blotting with specific antibodies. Total Histone H3 was used as a loading control. **b** RAW264.7 cells stably express scramble RNA or DOT1L shRNA collected for real-time polymerase chain reaction and western blot analyses. DOT1L mRNA and protein levels in DOT1L–knocked-down RAW264.7 cells are downregulated to half the levels in the control. H3K79me2 levels are significantly reduced in *DOT1L*–knocked-down RAW264.7 cells compared with the control. **c** Murine RAW264.7 cells stably express scramble RNA or DOT1L shRNA stimulated with 10 ng/mL RANKL for 60 h. Cells were fixed and stained using a TRAP kit. Representative images are shown. **d–f** OC number and surface area measurements are presented according to TRAP staining results shown in **c**. **g** Bone resorption assay: RAW264.7 cells stably express scramble RNA or DOT1L shRNA stimulated with 10 ng/mL RANKL for 5 days in osteoassay plates. Representative images of toluidine blue staining are shown. **h** Percentage of resorption area relative to the total area of the osteoassay plate calculated on the basis of staining results shown in **g**. Experimental data are expressed as mean ± standard deviation.*P <  0.05, ****P <  0.0001, two-tailed unpaired t-test, compared with the scramble control

To investigate changes in DOT1L expression and activity during OC differentiation, a RANKL-induced osteoclastogenesis model of the mouse macrophage cell line RAW264.7 was used. The immunoblotting results revealed that during OC differentiation, dimethylation on H3K79 (H3K79me2) dramatically increased and was accompanied by the upregulation of OC-specific proteins NFATc1, TRAP, and CTSK. The level of the H3K79 methyltransferase DOT1L increased 24 h before the H3K79me2 transcription burst (Fig. 1a).

To determine the influence of DOT1L on OC differentiation, we knocked down DOT1L in RAW264.7 cells and induced OC differentiation by RANKL stimulation. Knockdown of DOT1L downregulated the level of H3K79me2 (Fig. 1b). During osteoclastogenesis, these cells showed increased OC surface area, large OC size (syncytia with >20 nuclei) (Fig. 1c–e), and enhanced resorption activity compared to scramble negative control cells (Fig. 1g–h). However, the total number of OCs remained unchanged (Fig. 1f).

### DOT1L inhibitors enhance cell fusion and resorption activity in RAW264.7 osteoclastogenesis model

To assess if the role of DOT1L in OC differentiation is dependent on its enzymatic activity, two DOT1L inhibitors—EPZ004777 and EPZ5676—were used in a RAW264.7 osteoclastogenesis model.

The specificity of EPZ004777 and EPZ5676 inhibitory activity in RAW264.7 cells and RAW264.7-derived OCs was assessed by western blotting. Although both inhibitors led to a concentration-dependent decrease in global H3K79 dimethylation, EPZ5676 showed greater potency in DOT1L inhibition than EPZ004777 (Fig. S1). The methylation levels at seven other sites did not decrease. In contrast, in RAW264.7 cells, H3K27me2 and H3K36me2 levels were elevated, while in OCs, H3K36me2 levels were increased slightly. This suggests that EPZ5676 and EPZ004777 selectively inhibit DOT1L and indirectly lead to an upregulation of H3K27me and H3K36me levels (Fig. S1).

**Fig. 2:**
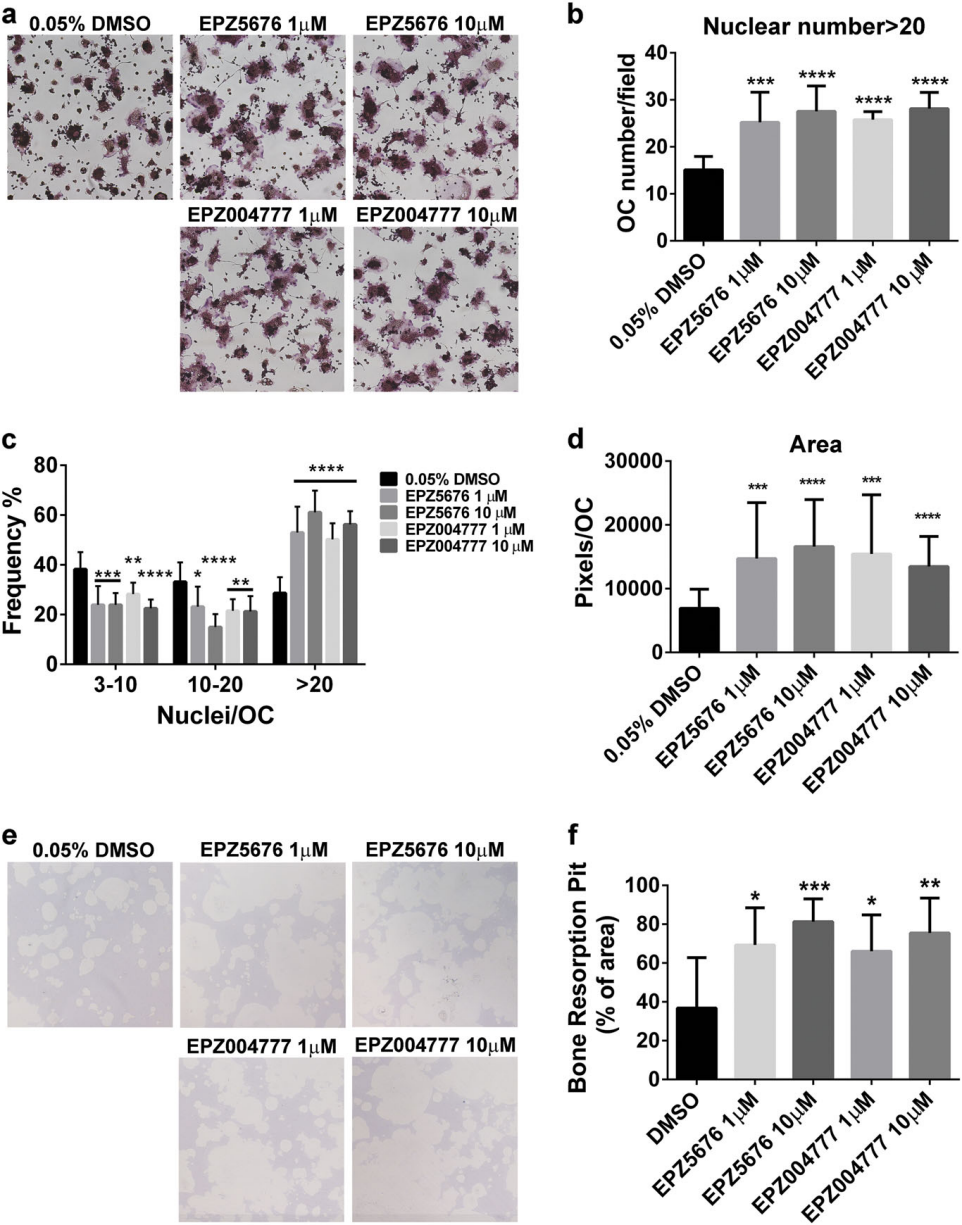
DOT1L enzyme inhibition enhances OC fusion and resorption ability **a** RAW264.7 cells pretreated with DMSO or the indicated concentrations of DOT1L inhibitors (EPZ5676 and EPZ004777) and stimulated with RANKL for 60 h. OCs were fixed and stained for TRAP. **b–d** OC number and surface area measurement according to TRAP staining results shown in **a**. **e** Bone resorption assay: OCs were treated with DOT1L inhibitor or DMSO for 5 days in osteoassay plates. Representative images of toluidine blue staining are shown. **f** Percentage of resorption area relative to the total area of osteoassay plates calculated on the basis of staining results shown in **e**. Experimental data are expressed as mean ± standard deviation. *P < 0.05, **P < 0.01, ***P < 0.001, ****P < 0.0001, two-tailed unpaired *t*-test, compared with the DMSO treatment. DMSO: dimethyl sulfoxide.

Furthermore, treatment with DOT1L inhibitors increased the proportion and number of large OCs, which was approximately twice that observed in the control group (Fig. 2a–c), as well as the cell surface area (Fig. 2d). However, the total number of OCs remained largely unaffected (Fig. S2). Furthermore, no significant difference was observed between the effects of treatment at 1 and 10 µM of the DOT1L inhibitors. In bone resorption assays, both EPZ5676 and EPZ004777 increased the resorption pit area at 1 and 10 µM concentrations (Fig. 2e–f).

### DOT1L inhibition enhances bone loss in ovariectomized (OVX) mice

**Fig. 3:**
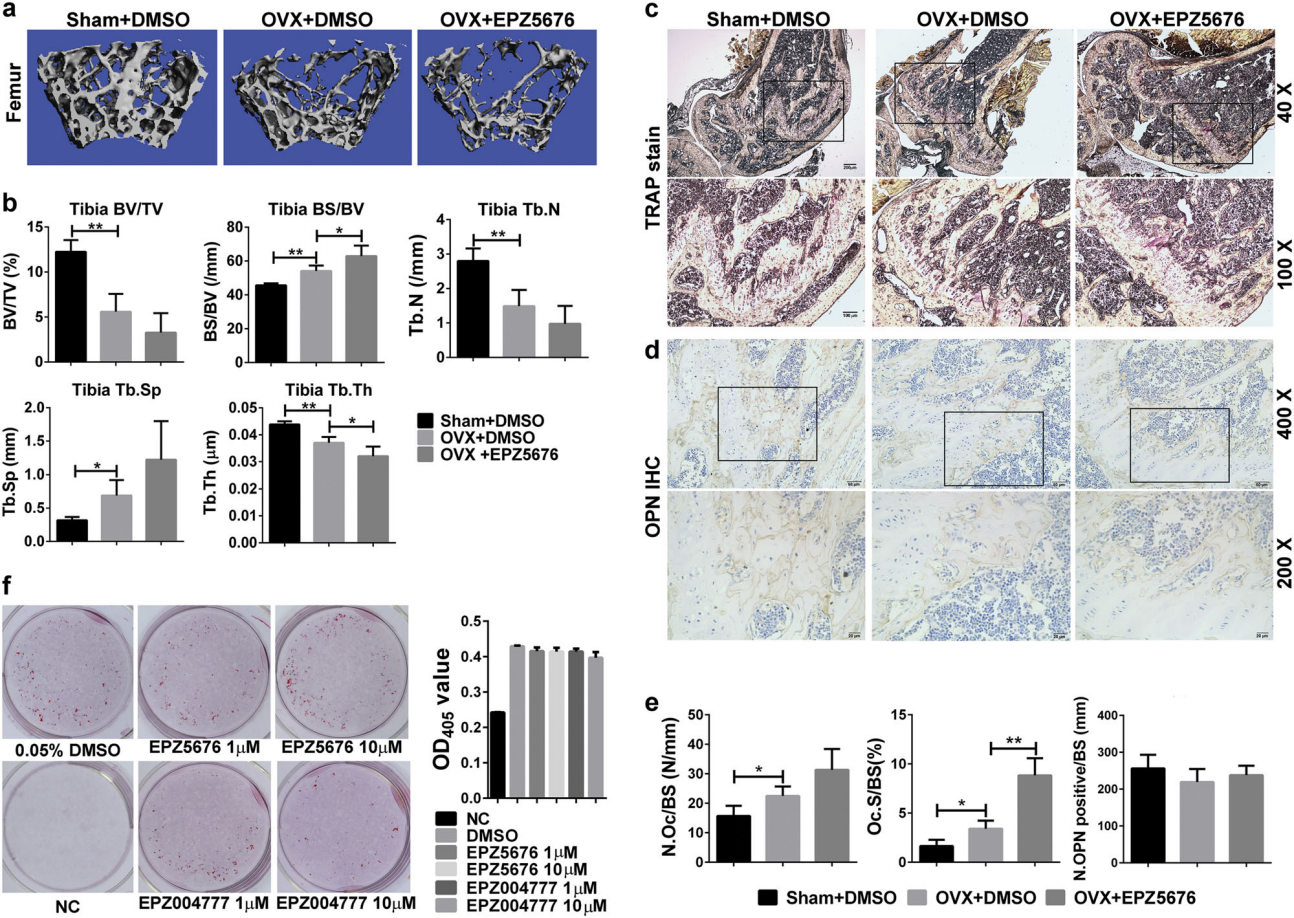
Inhibition of DOT1L aggravates bone mass reduction in OVX mice **a** OVX mice were sacrificed after 8 weeks of EPZ5676 treatment. Micro-computed tomography images (*n* = 5) of trabecular bone from femurs in the sham-operated group (Sham), OVX mice, and EPZ5676-treated OVX mice. **b** Measurements of the ratios of bone volume to total volume (BV/TV), bone surface to bone volume (BS/BV), trabecular number (Tb.N), trabecular spacing (Tb.Sp), and trabecular thickness (Tb.Th) for the indicated mice. **c** For static histomorphometric analysis, femurs were fixed and embedded in paraffin. The paraffin-embedded bone sections from Sham, OVX, and EPZ004777-treated OVX mice were double-stained with TRAP and hematoxylin (top, low magnification [40 × ] of proximal femoral metaphysis; bottom, high magnification (100 × ) of the black frame in the top panel). **d** Immunohistochemical staining of osteoblastic markers OPN in femurs from Sham, OVX, and EPZ004777-treated OVX mice. **e** OC surface/bone surface (Oc.S/BS, %), and OC number/bone surface (N.Oc/BS, N/mm). Number of OPN-positive (N.OPN^+^) osteoblasts on the bone surface (N.OPN positive/BS, N/mm) was calculated. **f** MC3T3E1 was induced to differentiate into osteoblasts by treatment with 50 µg/mL l-ascorbic acid and 10 mM β-glycerophosphate in the presence of control or DOT1L inhibitors for 18 days and then fixed with Alizarin S red. Left panel shows Alizarin S red staining. Right panel shows the optical density at 405 nm of the Alizarin S red extract from the left panel. Experimental data are expressed as mean ± standard deviation. *P < 0.05, **P < 0.01, ***P < 0.001, ****P < 0.0001, two-tailed unpaired *t*-test. OPN: osteopontin.

The RAW264.7 model data showed that DOT1L inhibition enhanced OC fusion and bone resorption. To further validate this finding *in vivo*, OVX mice received subcutaneous implants of osmotic pumps delivering EPZ5676. Compared to vehicle treatment, EPZ5676 treatment significantly decreased bone mass in the femur (Fig. 3a), increased trabecular bone surface to bone volume (BS/BV), and decreased trabecular thickness (Tb.Th) (Fig. 3b). TRAP staining showed that EPZ5676 treatment increased OC surface area *in vivo*, without a change in the OC number (Fig. 3c–e). DOT1L inhibitors did not affect osteoblast differentiation *in vivo* and *in vitro* (Fig. 3d–f).

### DOT1L inhibition alters different genes in RANKL-induced and untreated RAW264.7 cells

**Fig. 4:**
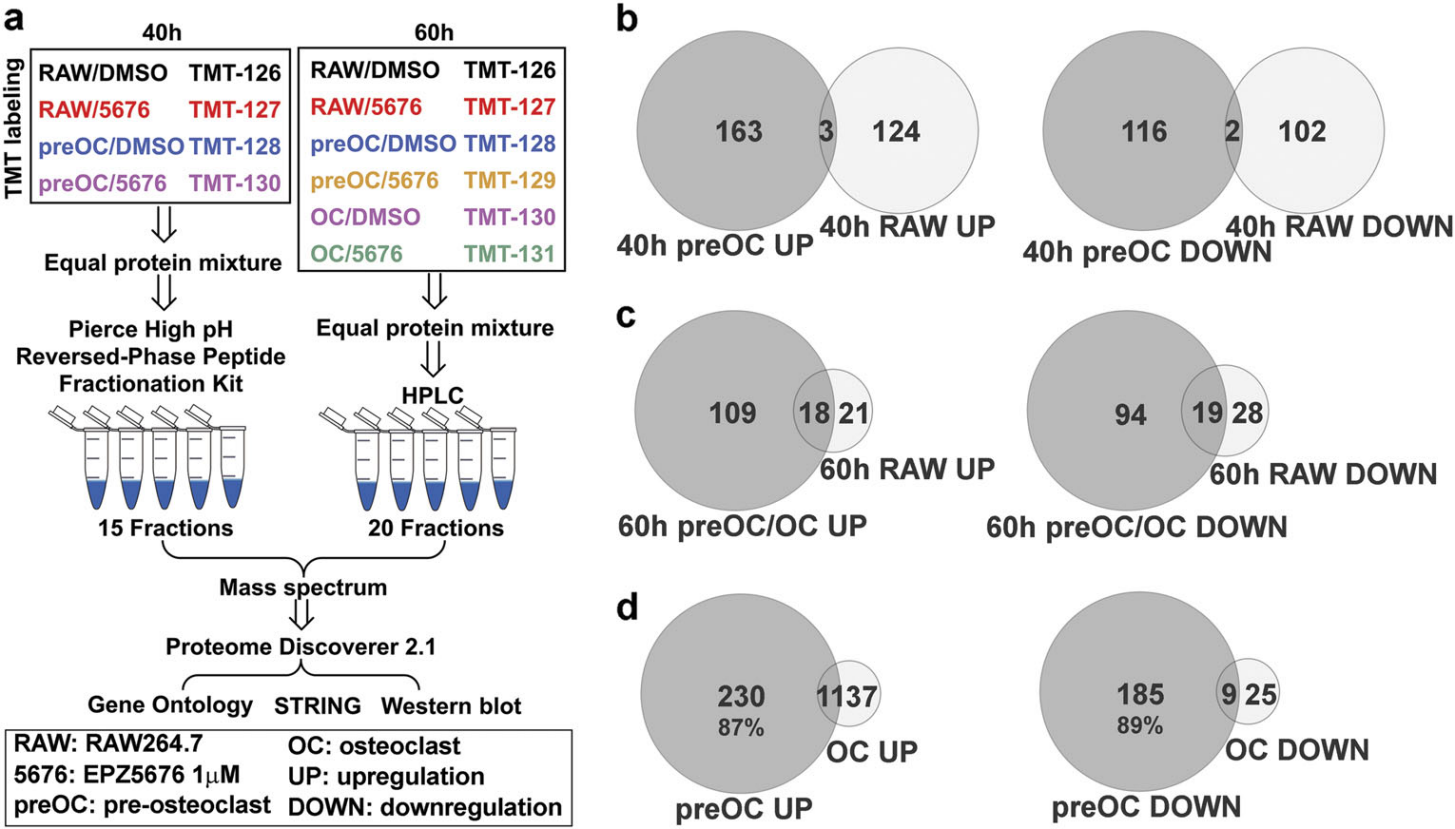
DEPs associated with DOT1L inhibition are related to OC differentiation, mainly in pre-OCs RAW264.7 cells pretreated with DMSO or the DOT1L inhibitor EPZ5676 were stimulated with RANKL for the indicated times. RAW264.7 cells pretreated with DMSO or the DOT1L inhibitor EPZ5676 for the indicated times without RANKL stimulation were used as controls. **a** Experimental flowchart of tandem mass tag-based quantitative proteomics and subsequent analysis. **b** Venn diagram of DEPs in RAW264.7 cells and pre-OCs after 40-h treatment. **c** Venn diagram of DEPs in RAW264.7 cells and pre-OCs/OCs after 60-h treatment. **d** Venn diagram of DEPs in pre-OCs (40-h and 60-h) and OCs. DEPs, differentially expressed peptides

To identify the proteins regulated by DOT1L in osteoclastogenesis, we used mass spectrometry-based quantitative proteomic tools. In the RAW264.7 osteoclastogenesis model, RAW264.7 cells were also called pro-osteoclasts, 40-h RANKL-induced RAW264.7 cells were considered as pre-OCs, and 60-h RANKL-induced RAW264.7 cells are a mixed population of pre-OCs and OCs. As shown in Fig. 4a, RANKL-induced and untreated RAW264.7 cells were collected with or without DOT1L inhibitor treatment. Two groups, one for 40-h endpoint and the other for 60-h endpoint, were set up for tandem mass tag (TMT)-based quantitative proteomic analysis. The identified proteins are listed in Table S1.

With a threshold of >1.2-fold change or <0.85-fold change, EPZ5676-induced differentially expressed proteins (DEPs) were identified. We found that the EPZ5676-induced DEPs of RANKL-induced RAW264.7 cells (pre-OCs and OCs) were remarkably different from those of untreated RAW264.7 cells (Fig. 4b, c), which indicated that DOT1L regulates a group of OC differentiation-associated proteins. In addition, more than 85% of the OC differentiation-related DEPs (OC-DEPs) were in pre-osteoclasts (Fig. 4d), suggesting that DOT1L mainly acts in the early pre-osteoclast stage.

### Pre-OC stage DOT1L-regulated proteins are involved in regulation of OC formation and viability, while the OC stage proteins in bone-resorption

To clarify the role of DOT1L-regulated proteins in OC differentiation, gene ontology (GO) and Kyoto Encyclopedia of Genes and Genomes (KEGG) analysis were used to analyze the biological function and signaling pathways of OC-DEPs.

**Fig. 5:**
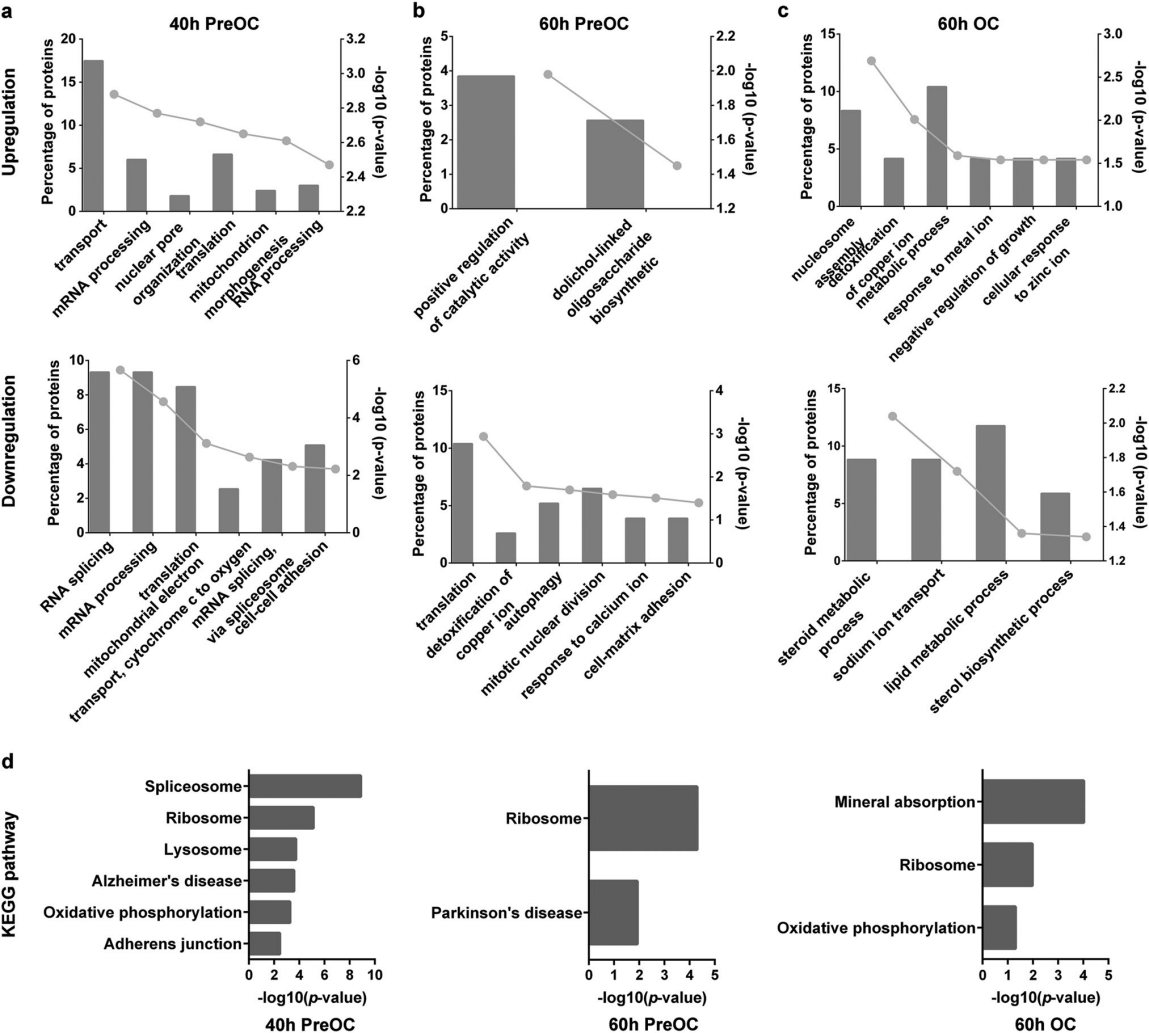
GO biological processes and KEGG pathways analysis of DOT1L inhibitor-induced OC-related DEPs DEPs were identified via proteomic approaches based on tandem mass tag-labeling in three stages of OC differentiation induced by DOT1L inhibitor for the indicated times: 40-h pre-OCs, 60-h pre-OCs, and 60-h OCs. DEPs were submitted to the DAVID online GO classification system and KEGG pathway analysis (http://david.ncifcrf.gov/). The enrichment significance (-log10 [*P*-value]) threshold is *P* < 0.05. The six most-enriched categories are shown if more than six categories exceed the threshold. **a–c** Analysis of upregulated proteins and downregulated proteins in **a** 40-h pre-OCs, **b** 60-h pre-OCs, and **c** 60-h OCs. **d** KEGG pathway analysis of DEPs in 40-h pre-OCs, 60-h pre-OCs, and 60-h OCs. GO: gene ontology, KEGG: Kyoto Encyclopedia of Genes and Genomes, DEPs: differentially expressed proteins

Among the DOT1L inhibitor-induced OC-DEPs in 40-h pre-OC (Fig. 5a), both up- and downregulated proteins were found to function in RNA processing, mRNA processing and translation processes, which are important for protein biosynthesis. The upregulated proteins participate in transport and nuclear pore organization, which are related to import and/or export of proteins or mRNA to the nucleus. This finding provides a molecular basis for the production of large amounts of protein in OC differentiation. Furthermore, proteins in energy generation-associated processes such as mitochondrion morphogenesis and mitochondrial electron transport were altered, providing energy support for OC differentiation. The downregulation of protein expression in the cell–cell adhesion process changes the migration ability of pre-OCs. In conclusion, OC-DEPs in 40-h pre-OCs participated in biological processes which are closely related to the formation of OCs.

Among the DOT1L inhibitor-induced OC-DEPs in 60-h pre-OCs (Fig. 5b), upregulated proteins were involved in catalytic regulation and oligosaccharide biosynthetic processes, which are important for vesicle trafficking. In contrast, downregulated proteins were related to cytotoxic resistance such as copper ion detoxification, autophagy, and response to calcium ions. This finding suggests that DOT1L may affect the viability of pre-OCs.

Among the DOT1L inhibitor-induced OC-DEPs in 60-h OCs (Fig. 5c), upregulated proteins were associated with metabolic and cytotoxic resistance processes such as nucleosome assembly, negative regulation of growth, response to metal ions, and copper ion detoxification. In contrast, downregulated proteins were enriched in metabolic processes such as steroid metabolism, lipid metabolism, and sterol biosynthetic processes. These biological processes are associated with OC bone resorption.

The DOT1L inhibitor-induced OC-DEPs in pre-OCs were grouped in OC differentiation-associated pathways such as lysosome and oxidative phosphorylation. The DOT1L inhibitor-induced OC-DEPs in OCs were enriched in the OC resorption-related pathway of mineral absorption (Fig. 5d). These findings suggest that DOT1L inhibitor-induced OC-DEPs in pre-OCs are involved in the regulation of OC formation and viability and that mature OCs are involved in the regulation of bone resorption.

### DOT1L inhibition alters the levels of proteins related to electron transport chain, autophagy, migration, and adhesion in pre-OCs and upregulates CD9 and MMP9 in OCs

**Fig. 6:**
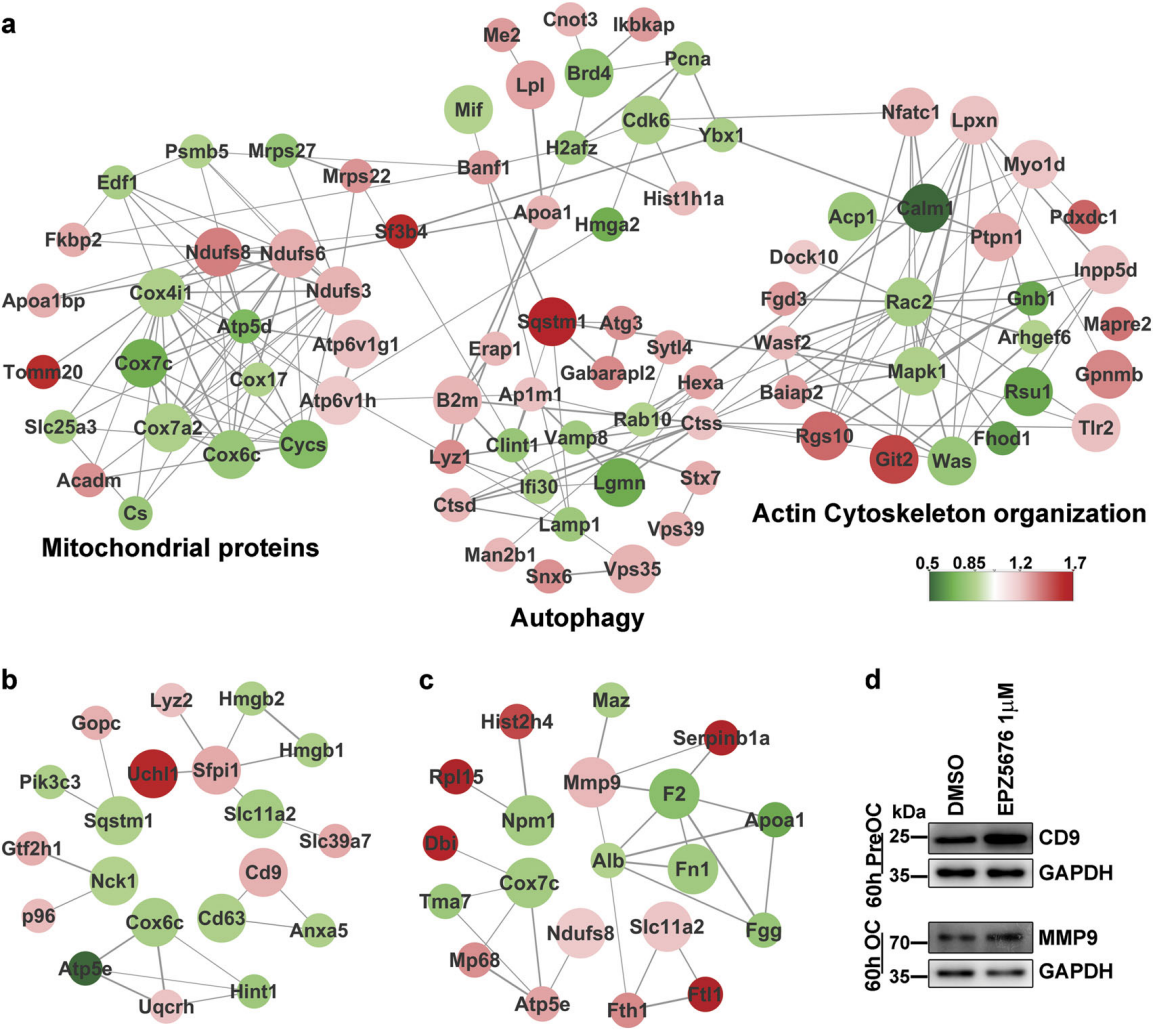
Interaction network constructed with OC-associated DEPs in three stages of OC formation Proteins previously reported to be associated with OC differentiation (indicated by the large circle) and their first connected nodes (indicated by the small circle) were extracted from Fig. S3. The width of the lines connecting the proteins indicates the connection score obtained from the database. Up- or downregulation is indicated by the color of nodes (red, upregulated; green, downregulated). **a–c** Network of OC-associated DEPs identified in 40-h pre-OCs, 60-h pre-OCs, and 60-h OCs. Function groups were classified on the basis of UniProt function and published reports. Panels **a**–**c** share one color ruler. **d** Verification of upregulation of protein expression by western blotting. Upregulation of CD9 in 60-h pre-OCs and MMP9 in 60-h OCs after DOT1L inhibition was detected in proteomic data and verified by western blotting using specific antibodies. DEPs: differentially expressed proteins

To identify the key molecules regulated by DOT1L that affect OC formation and OC resorption, protein–protein interaction networks of DOT1L inhibitor-induced OC-DEPs were constructed using Cytoscape 3.4 with the STRING App (Fig. S3). Most OC-DEPs involved in OC differentiation, mainly mitochondrial proteins, autophagy-related proteins, and cytoskeletal regulatory proteins, were enriched at the 40-h pre-OC stage (Table S3) (Fig. 6a).

Among the mitochondrial proteins, ten proteins were components of the electron transport chain (Fig. 6a, Fig. S4). Three upregulated proteins—Ndufs3, Ndufs6, and Ndufs8—were components of Complex I (NADH-ubiquinone oxidoreductase). Cytochrome c (Cycs); Complex V (ATP Synthase F1 Complex) protein Atp5d; and five proteins in Complex IV (cytochrome C oxidase), i.e., Cox4i1, Cox6c, Cox7a2, Cox7c, and Cox17 (AI037035) were downregulated. However, there were no changes in the expression of other proteins detected in Complex I–V (Fig. S4).

Following EPZ5676 treatment, the majority of OC-DEPs involved in autophagy were upregulated (Fig. 6a), including SNAREs (Snx6, Vps35, Vps39, and Stx7), lysosomal molecules (Ctsd, Ctss, Lyz1, B2m, Ap1m1, and Man2b1), autophagosome assembly proteins (Atg3 and Atg8-like Gabarapl2), and autophagy receptors (Sqstm1). Among these proteins, SNAREs^13^, Sqstm1^14^, and Atg3^15^ are central proteins in autophagy, whereas Sqstm1, B2m, Vps35, and Lgmn participate in OC differentiation (Table S3).

Proteomic analysis of the 40-h pre-OCs revealed 24 DEPs involved in cytoskeleton organization (Fig. 6a). Most of these DEPs (16 of 24, Table S4) were involved in regulation of Rho-GTPases, with approximately equal numbers of upregulated and downregulated DEPs. The remaining eight DEPs involved in actin binding (Lpxn and Myo1d), cell adhesion (Pdxdc1, Mapre2, and Inpp5d), and migration (NFATc1, Tlr2, and Gpnmb) were upregulated. Six proteins, Lpxn, NFATc1, Gpnmb, Inpp5d, Mapre2, and Tlr2, have been reported to play a positive role in cell migration and adhesion regulation^16–21^. In addition, NFATc1 upregulation was verified by western blotting (Fig. 8b).

Most proteins in the unnamed clusters are chromatin remodeling-related proteins, including CDK6, H2afz, Banf1, Hmga2, Hist1h1a, Ybx1, Pcna, and Brd4. In the unnamed protein clusters, CDK6 and H2afz appear to be connected through Banf1, Apoa1, and Ybx1 with the three main clusters shown in Fig. 6a.

**Fig. 7:**
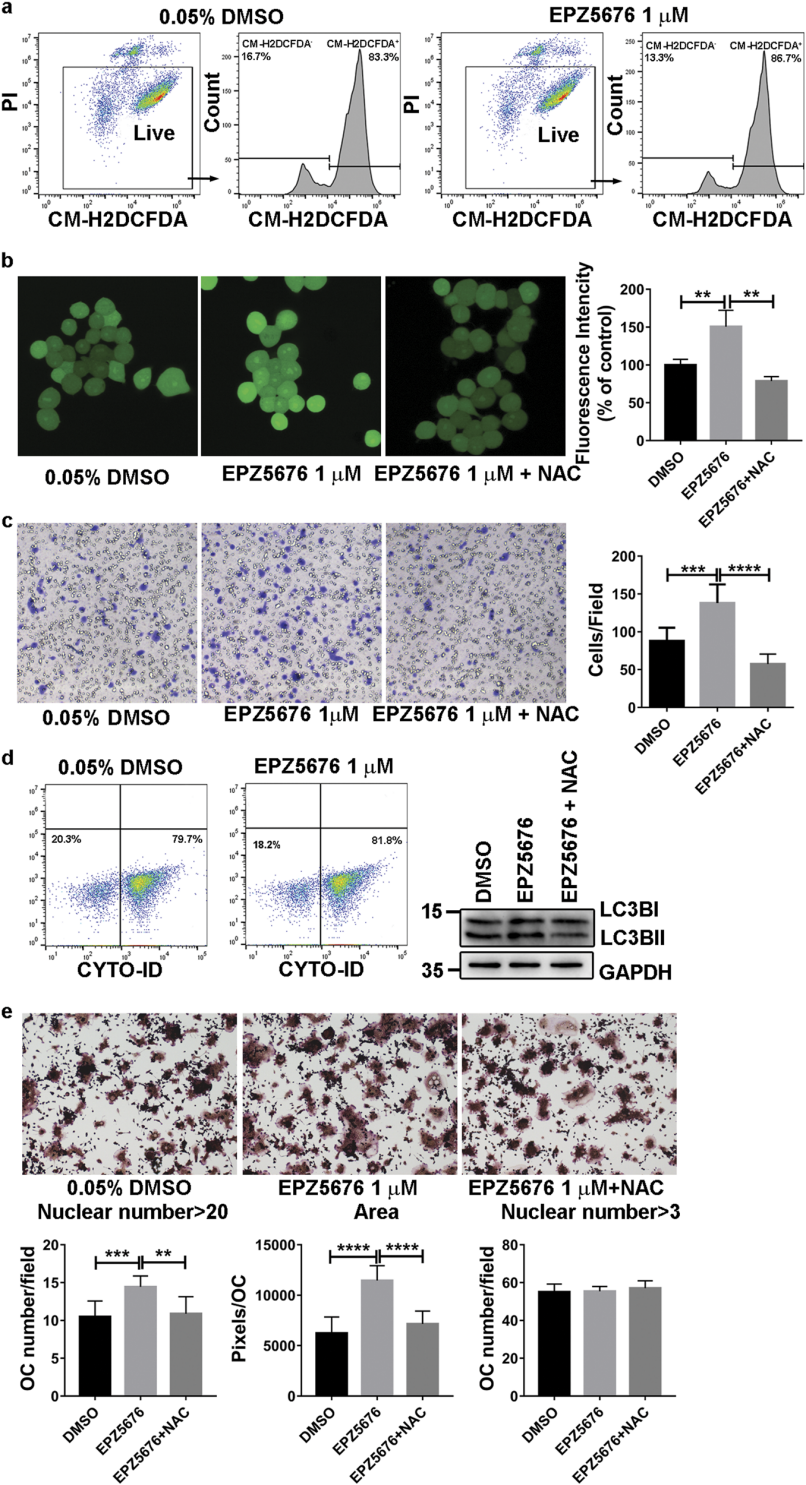
DOT1L inhibition increases ROS generation, autophagy flux, and migration ability of 40-h pre-OCs RAW264.7 cells pretreated with DMSO or the DOT1L inhibitor EPZ5676 (1 µM) were stimulated for indicted time with RANKL. **a** Flow cytometric analysis of ROS generation in 40-h pre-OCs by PI and CM-H2DCFDA double-staining. PI-negative live cells were gated for CM-H2DCFDA measurement. **b** Confocal fluorescence microscopy detection of ROS generation in 40-h pre-OCs treated with DMSO, EPZ5676 (1 µM) or EPZ5676 (1 µM) combined with NAC (1 mM). The fluorescence intensity was calculated with ImageJ software (**c**) Migration analysis of 40-h pre-OCs using transwell migration assays. Cell number per field was counted. Experimental data are expressed as the mean ± standard deviation. **d** Flow cytometric analysis of autophagy in 40-h pre-OCs using the CYTO-ID autophagy detection kit. **e** TRAP staining and analysis of OCs treated with DMSO, EPZ5676 (1 µM) or EPZ5676 (1 µM) combined with NAC (1 mM). ***P < 0.001, two-tailed unpaired *t*-test, compared with DMSO treatment. ROS: reactive oxygen species, DMSO: dimethyl sulfoxide, CM-H2DCFDA: chloromethyl derivative of 2’,7’-dichlorodihydrofluorescein diacetate, PI: propidium iodide.

Live cells (propidium iodide (PI)-negative) were analyzed for reactive oxygen species (ROS) generation by chloromethyl derivative of 2′,7′-dichlorodihydrofluorescein diacetate (CM-H2DCFDA) staining. The percentage of CM-H2DCFDA-positive 40-h pre-OCs increased from 83.3% to 86.7% after EPZ5676 treatment (Fig. 7a). And the fluorescence intensity, which indicates ROS generation was significantly enhanced by EPZ5676 (Fig. 7b). After EPZ5676 treatment, the migration ability of 40-h pre-OCs measured in Transwell assays increased significantly (Fig. 7c). Autophagy flux, indicated by the CYTO-ID autophagy detection assay, increased from 79.7% to 81.8% after EPZ5676 treatment of 40-h pre-OCs compared with the control cells. The LC3BII/BI expression level also slightly increased after EPZ5676 treatment of 40-h pre-OCs compared with the control cells (Fig. 7d). When ROS were inhibited with 1 mM *N*-acetyl cysteine (NAC), EPZ5676-induced OC differentiation, autophagy activation, and cell migration enhancement was attenuated (Fig. 7b–e).

Although there was no significant functional clustering of 60-h pre-OC and 60-h OC DEPs, several proteins including CD9 and MMP9 were associated with OC fusion and resorption. Among the 60-h pre-OC DEPs, CD9, a positive regulator of OC fusion^22^, was upregulated (Fig. 6b, d). Among the 60-h OC DEPs, MMP9^23^, an OC resorption protein, was upregulated by EPZ5676 treatment (Fig. 6c–d).

### Inhibitor treatment enhance NFATc1 and NF-κB transcription activation

**Fig. 8:**
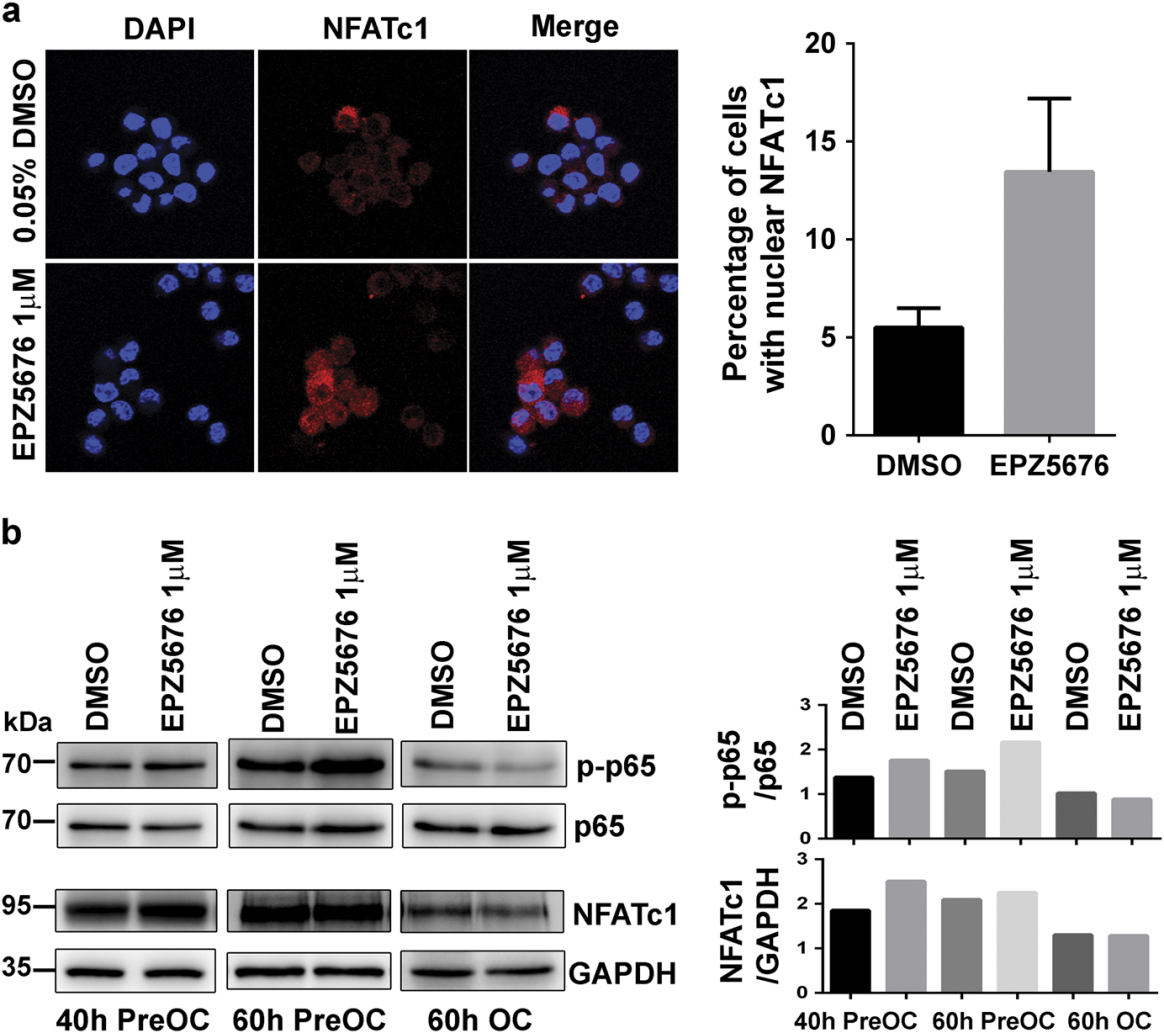
Inhibition of DOT1L promotes NFATc1 and NF-κB (p-65) nuclear translocation **a** Immunofluorescence analysis of NFATc1 nuclear translocation. RAW264.7 cells were treated with DMSO or 1 µM EPZ5676 for 40 h. Red color indicates NFATc1 expression, as seen under a fluorescence microscope, and the nuclei are stained blue. Representative results of the two replica experiments are shown (magnification, ×600). **b** Murine RAW264.7 cells treated with DOT1L inhibitor or DMSO were stimulated with 10 ng/mL RANKL for the indicated time. The 40-h pre-OCs (40-h PreOC), 60-h pre-OCs (60-h PreOC), and 60-h OCs (60-h OC) were collected for western blot analysis. Phosphorylation of p65 on S536 and NFATc1 expression were detected with specific antibodies; grayscale analysis was conducted using p65 and glyceraldehyde 3-phosphate dehydrogenase (GAPDH) as the internal controls, respectively. Experimental data are expressed as mean ± standard deviation. DMSO: dimethyl sulfoxide.

NFATc1 and NF-κB are transcriptional regulators that control OC differentiation by affecting the expression of many proteins, including TRAP, CTSK, and MMP9. We next evaluated whether DOT1L enhanced the transcriptional activities of these proteins. We found that the nuclear translocation ratio of NFATc1 increased approximately threefold following 40-h EPZ5676 treatment of RAW264.7 cells (Fig. 8a). At the same time, the expression of NFATc1 increased in 40-h pre-OCs and remained unchanged in 60-h pre-OCs and OCs after EPZ5676 treatment. The phosphorylation (S536) level of RELA (also known as p65) also increased in 40-h pre-OCs and 60-h pre-OCs compared with the control (Fig. 8b). The upregulation of NF-κB phosphorylation and NFATc1 translocation observed in our study indicates transcriptional activation^24^^,^^25^, which changes the expression of its downstream proteins. This finding suggests that DOT1L influences NFATc1 nuclear translocation, NF-κB phosphorylation activation, and indirect regulation of OC-DEPs by H3K79me2.

## Discussion

In this study, we used RAW264.7-derived OCs to determine the function of DOT1L in osteoclastogenesis. To our knowledge, this is the first study to show that DOT1L expression is upregulated in RANKL-induced OCs and that knockdown of *DOT1L* or enzymatic inhibition of DOT1L increases OC size and enhances resorption ability. DOT1L acts as a negative regulator of OC differentiation. The elevated DOT1L expression may be involved in controlling OC differentiation to balance OC bone resorption activity and osteoblast bone formation. The consistency of the effects of *DOT1L* knockdown and DOT1L enzymatic inhibition on OC differentiation indicate that its effects on OCs are mediated mainly through its methyltransferase catalytic activity.

This study did not ascertain whether DOT1L-regulated OC-DEPs are regulated by H3K79me directly or indirectly. We speculate that both regulation pathways exist simultaneously. Firstly, DOT1L inhibition could active the transcriptional activity of NFATc1 and NF-κB, which are the key regulators response to OC differentiation. In addition, we observed that the DOT1L inhibitors EPZ5676 and EPZ004777 were also able to upregulate the levels of H3K27me and H3K36me, which are involved in the regulation of OC differentiation^6,7,9^. Thus, the regulatory effect of DOT1L on OC differentiation may also include indirect regulation.

DOT1L inhibitors enhanced bone erosion in the OVX mouse model, suggesting that decreased DOT1L level or impaired enzyme activity may decrease bone density in menopausal women. The relationship between DOT1L expression levels in bone tissue or serum and postmenopausal osteoporosis (OP) requires further investigation. Our results also indicate that the DOT1L protein network may be a therapeutic target for OP. Many of the DOT1L connected proteins have been reported to be involved in the regulation of OC differentiation (Table S3) and some, such as Rho GTPase proteins, are the targets of drugs for the clinical management of OP^26^. Bone homeostasis is maintained by a balance between bone resorption by OCs and bone formation by osteoblasts^27^. In this study, EPZ5676 enhanced OC differentiation, but did not affect osteoblast differentiation *in vivo* and *in vitro*(Fig. 3d–f), suggesting that DOT1L affects bone homeostasis mainly through OC regulation.

It has been reported that DOT1L protects against osteoarthritis (OA)^12^. Our study indicates that DOT1L also prevents OP, which suggests that DOT1L may be one of the key factors involved in OP and OA. OC hyperactivation is also another common factor involved in OP and OA^5,28^. In combination with our observation that DOT1L inhibition enhances OC differentiation and resorption in the present study, we hypothesize that regulation of OC by DOTL1 may play roles in both diseases. This possibility provides a new insight into the relationship between OP and OA. The previous study showing that DOT1L protects against OA focused on its role in cartilage homeostasis maintained by chondrocytes^11,12^. Our results indicate that OC activation regulated by DOT1L may also be involved in OA.

Fusion of mononuclear pre-OCs plays a vital role in the maturation of OCs^22,29^. DOT1L inhibitor treatment increased the number and surface area of large OCs compared with the control (Fig. 2a–d), indicating that DOT1L inhibitors enhance cell fusion. Cell fusion is associated with cell migration and adhesion^30^. Among the 40-h pre-OC DEPs, six proteins, Lpxn, NFATc1, Gpnmb, Inpp5d, Mapre2, and Tlr2, are known to play a positive role in the regulation of cell migration and adhesion^16–21^. EPZ5676 treatment greatly enhanced cell migration in 40-h pre-OCs (Fig. 7c). Furthermore, 15 proteins influenced or participated in OC fusion (Table S3). These findings indicate that DOT1L inhibition enhances cell fusion by regulating cell migration and adhesion.

Treatment with DOT1L inhibitors was found to be associated with regulation of other factors related to OC differentiation and resorption capability, including ROS and autophagy. ROS are produced by and activate OCs^31–34^. In this study, CM-H2DCFDA staining showed a slight increase in the total cell ROS production after EPZ5676 treatment (Fig. 7a, b). This increase could have been caused by DOT1L inhibitor-induced electron transport chain disorders. The mitochondrial electron transport chain, which is critically involved in ROS production^35^, involves generation of electrons in Complex I and II, which then pass sequentially to coenzyme Q (ubiquinone), Complex III, Cycs, and Complex IV. Thus, the Complex I protein upregulation, unaffected Complex III protein expression, and Cycs and Complex IV downregulation (Fig. 6a, Fig. S4) observed in proteomics data induced by DOT1L inhibitor EPZ5676 may indicate an increased rate of electron production and reduced electron transport. Considering that inhibition of electron transfer from complex I to ubiquinone causes a profound increase in superoxide production^36^, we speculate superoxide production was elevated by altered electron transport chain protein expression. In addition, the levels of manganese-dependent superoxide dismutase, which is a scavenger of mitochondrial ROS^37^, remained unchanged (Table S1). This potentially resulted in increased ROS production by mitochondria, suggesting that EPZ5676 promotes OC differentiation by promoting ROS production.

Activation of autophagy significantly increases the number and mean size of multinucleated, TRAP-positive OCs^38^. In our study, the central autophagy proteins SNAREs^13^, Sqstm1^14^, VPS35^39^, and Atg3^15^ were upregulated in 40-h pre-OCs (Fig. 6a). Of these proteins, Sqstm1 and VPS35 have been reported to play roles in OC differentiation (Table S3), indicating increased autophagy activity, which was confirmed by the CYTO-ID autophagy detection assay and LC3BI/BII western blot (Fig. 7d). Thus, autophagy may be a target of DOT1L in the regulation of OC differentiation.

Wnt signaling is a well-known pathway involved in OC differentiation^40^ and the Wnt pathway genes Lef1, Tcf7, and Myc are regulated by DOT1L-mediated H3K79me^12^. However, Wnt pathway proteins were not detected in the proteomics data. To explore whether Wnt signaling mediated the effect of DOT1L on the DEPs identified in this study, especially ROS, autophagy and actin cytoskeleton organization-related proteins, we performed STRING analysis of the Wnt pathway proteins and DEPs (Fig. S5). We found that the Wnt pathway is closely associated with actin cytoskeleton organization-regulated proteins in a process that is mediated mainly by β-catenin (Fig. S5a). In 60 h preOCs, Myc and Axin act as linkers between the Wnt pathway and DEPs (Fig. S5b). In 60 h OCs, Myc and β-catenin mediate Wnt signaling and DEPs (Fig. S5c). This indicates that the Wnt pathway mediates the function of DOT1L in OCs, which is accordance with a previous report by Rossini et al.^41^.

In the unnamed protein cluster in Fig. 6a, Cdk6 was found to be a key molecule in determining the differentiation rate of OCs^42^ and appears to act as a connector of the three main clusters. CDK6 also connects with Hist1h1, which is an essential protein for chromatin structure formation. It has been reported that Hist1h1 overexpression induces chromosome condensation^43^, which plays roles in cell differentiation^44,45^ and suggests that Hist1h1 may regulate cell differentiation. In our results, Hist1h1 was upregulated by DOT1L inhibition, implying that Hist1h1 participates in OC differentiation by altering the chromatin condensation state.

In conclusion, we report a new regulator of OC differentiation—DOT1L. Our study showed that inhibition of DOT1L promotes OC fusion and resorption and enhances bone erosion *in vivo* mainly by its effect on OCs. Mechanistically, DOT1L inhibition increases ROS generation, autophagy activity, and pre-OC migration by disrupting expression of associated proteins. In addition, DOT1L inhibition increased NFATc1 nuclear translocation and NF-κB activation and strengthened OC fusion and expression of the resorption-related protein CD9 and MMP9. These findings implicate DOT1L as a novel therapeutic target for OC dysregulation-induced disease.

## Materials and methods

### OC differentiation, TRAP staining, and bone resorption assay

For TRAP staining, RAW264.7 cells were cultured in α-MEM (Hyclone, IL, USA) supplemented with 10% FBS (Gibco, MA, USA) and 10 ng/mL RANKL (R&D Systems, MN, USA) for 60 h. The cells were washed three times with PBS and TRAP staining was performed using Acid Phosphatase, Leukocyte (TRAPS) kits (Sigma Aldrich, MO, USA) following the manufacturer’s protocol. For bone resorption assays, RAW264.7 cells were seeded on Osteo Assay Surface (Corning, New York, USA) and stimulated with RANKL for 5 days. After scraping away the cells, the Osteo Assay Surface was stained with 5% toluidine blue. At least nine fields of view covering the center and periphery of the plate were assessed in each well and the mean cell number or area per field ( ± standard deviation) were calculated.

### In vitro osteoblastic differentiation

MC3T3E1 cells were cultured in α-MEM suppled with 10% FBS. For osteoblast differentiation, cells were plated at a density of 1.5 × 10^5^ cells per well in 6-well plate and induced with 50 µg/mL l-ascorbic acid (LAA, Sigma Aldrich, MO, USA) and 10 mM beta-glycerophosphate (β-GP, Sigma Aldrich, MO, USA). Medium was changed every 2 days. Alizarin red staining was performed after differentiated for 18 days.

### Lentivirus-mediated knockdown of DOT1L

A plasmid bearing DOT1L-specific shRNA (5ʹ-GTTTACACAGCTTCAATGATG-3′) were constructed using the lentivirus-derived vector pLKO.1-shRNA (Era Biotech, Shanghai, China). Scramble sequence (5ʹ-CAACAAGATGAAGAGCACCAA-3′) was used as a negative control. HEK293T cells were used for lentivirus packaging. Co-transfection of lentivirus vector and helper plasmids pCMV-VSV-G, pCMV-Gag-Pol, and pRSVRev (Era Biotech, Shanghai, China) was completed using Lipofectamine 2000 (Invitrogen, MA, USA) according to the manufacturer’s instructions. Viral particles were harvested at 48 and 72 h post-transfection and mixed ready for infection. Cells were transduced with the viral particles in the presence of 8 µg/mL polybrene. Stable RAW264.7 cells were selected by the addition of 1 μg/mL puromycin to the culture medium.

### Immunoblot assay

Western blot analysis was performed using the following Abs: DOT1L (ab157199), TRAP (ab191406), CTSK (ab19027), MMP9 (ab137867), CD9 (ab92726), GAPDH (M171-3), H3K79me2 (ab3594), H3K79me1 (ab2886), H3K9me3 (ab8898), H3K9me2 (ab1220), asymmetric H3R17me2 (ab8284), Pan H3 (ab1791), H4K20me3 (ab9053), H4K20me2 (ab9052), symmetric H4R3me2 (ab5823), and Pan H4 (ab10158) (all purchased from Abcam (Cambridgeshire, UK). p-p65 (3033), p65 (8242), H3K36me3 (9763), H3K36me2 (2901), H3K27me3 (9733), H3K27me2 (9728), and H3K4me3 (9751) were purchased from Cell Signaling Technology (MA, USA). NFATc1 (sc-7294) was purchased from Santa Cruz (California, USA).

### Quantitative real-time PCR

First stand cDNA was synthesized with the PrimeScript™ 1st strand cDNA Synthesis Kit (TaKaRa, CA, USA) using mRNA extracted with TRI reagent (Sigma Aldrich, MO, USA) as the template. Real-time PCR was performed with cDNA, primers, and Fast SYBR Green Master Mix (Thermo Fisher Scientific, MA, USA). GAPDH primer sequences: forward 5'-GTCGGTGTGAACGGATTTGG-3'; reverse, 5'-ATGTTAGTGGGGTCTCGCTC-3'. DOT1L primer sequences: forward 5'-CGAGGAAATCCCAGATCTCA-3'; reverse 5'-ATGGCCCGGTTGTATTTGT-3'.

### Immunofluorescence analysis

RAW264.7 cells were seeded on a glass slide placed in 6-well plate (Corning, New York, USA) at a density of 1.0 × 10^5^ cells/well and cultured for 40 h. After treatment with EPZ5676 (1 µM) or DMSO, RAW264.7 cells were fixed with 4% paraformaldehyde for 15 min and then permeabilized with 0.1% Triton X-100 in PBS for 5 min. Cells were then blocked for 1 h with 1% BSA in PADB buffer (1% BSA, 0.1% gelatin in PBS) and incubated with anti-NFATc1(sc-7294) in PADB buffer for 2 h; this was followed by an incubation with Alexa Fluor 594 secondary antibody (Thermo Fisher Scientific, MA, USA) for 1 h. Immunofluorescence images (600X) were captured with Olympus FV1000MPE (Olympus, Tokyo, Japan).

### Estimation of reactive oxygen species by flow cytometry

The chloromethyl derivative of 2′, 7′-dichlorodihydrofluorescein diacetate (CM-H2DCF-DA) fluorescent probe is commonly employed and may react with several ROS including hydrogen peroxide, hydroxyl radicals and peroxynitrite. Because dead or dying cells produces ROS, PI staining was combined with CM-H2DCFDA to evaluate ROS production only by living cells, which are PI negative. The 40-h pre-OCs treated with EPZ5676 or DMSO were incubated with 10 μM CM-H2DCFDA for 30 min in the dark. PI (final concentration = 1 μg/mL) was added immediately before flow cytometry analysis. A total of 10,000 events were acquired for analysis. The cellular viability was assessed by PI staining. Living cells (PI-negative) were selected by FACS gating. PI fluorescence in the FL-3 channel (620 nm) and DCF fluorescence in the FL-1 channel (525 nm) were recorded by BD Accuri C6 (BD Biosciences, New Jersey, USA).

### Flow cytometric analysis of autophagic activity by Cyto-ID staining

The Cyto-ID fluorescent reagent specifically labels autophagic vacuoles and colocalizes with LC3. Thus, the Cyto-ID Autophagy Detection Kit (ENZO Life Sciences, Inc., NY, USA) was used to detect autophagosome formation in cells. Briefly, cells were washed twice with PBS, collected, and resuspended in 0.5 mL of freshly diluted Cyto-ID reagents in phenol-red-free culture medium containing 2% FBS. After incubation at 37 °C for 30 min, the Cyto-ID fluorescence of cells was analyzed immediately using BD Accuri™ C6 (BD Biosciences, New Jersey, USA). The percentage of Cyto-ID positive cells was used to assess the formation of autophagosomes.

### Transwell migration assay

Transwell inserts with 8-mm pore size (Corning, New York, USA) were placed in 24-well plates. To test the migration ability, 1 × 10^5^ 40-h pre-OCs in 100 μL of a serum-free medium were placed in the upper chamber, and 600 μL of the same medium containing 10% FBS was added into the lower chamber. The cells were incubated for 24 h. The cells on the upper side of the filters were removed with cotton swabs, then fixed in 3.7% formaldehyde for 30 min before staining with 0.05% crystal violet for 45 min. The cells on the underside of the filters were examined by light microscopy. At least nine fields of view covering the center and periphery of the plate were assessed in each well and the mean cell number per field (±standard deviation) was calculated.

### TMT-based quantitative proteomics

Proteins were dissolved in 8 M urea. Protein concentration was measured using Nano Drop 2000 (Thermo Fisher Scientific, MA, USA). Equal amounts of proteins from each sample was reduced with dithiothreitol (DTT) and alkylated with iodoacetamide (IAA) before dilution in 1 M urea. Proteins were digested in the presence of Trypsin/Lys-C (Promega, WI, USA) for 12 h. Digested proteins were labeled using the TMT Mass Tagging Kit (Thermo Fisher Scientific, MA, USA). The corresponding TMT molecules for the different samples are shown in Fig. 3a. After labeling, samples were pooled and dissolved in 0.1% trifluoroacetic acid (TFA).

### Peptide fractionation

Pooled TMT-labeled peptides of four samples in the 40-h DOT1L inhibitor treated cells were separated with Pierce High pH Reversed-Phase Peptide Fractionation Kit (Thermo Fisher Scientific, MA, USA). Flow through from 15 acetonitrile (ACN) gradients (5, 10, 12.5, 15, 17.5, 20, 22.5, 25, 27.5, 30, 32.5, 35, 40, 50, and 100%) was collected, dried, and resolved in 0.1% TFA for mass spectrometry analysis.

Pooled TMT-labeled peptides of six samples in 60-h DOT1L inhibitor treated cells were fractionated into 20 fractions with Xbridge BEH300 C18 column (Waters, MA, USA) on a Thermo UltiMate 3000 UPLC workstation (Thermo Fisher Scientific, MA, USA). Fractions were dried, and resolved in 0.1% TFA for Mass spectrometry analysis.

### Mass spectrometry analysis

Liquid chromatography–mass spectrometry/mass spectrometry (LC-MS/MS) was performed as described previously^46,47^ with slight modifications. The peptide mixture was separated with the EASY-nLC 1000 system equipped with a homemade fused silica capillary column (75 μm ID, 150 mm length; Upchurch, Oak Harbor, WA, USA) packed with C-18 resin (300 A, 5 μm; Varian, Lexington, MA, USA). Peptides eluted using a 120-min gradient at a flow rate 0.300 μL/min were ionized by online electrospray ionization in Thermo Orbitrap Fusion mass spectrometer (Thermo Fisher Scientific, MA, USA) in positive-ion mode. The MS data were collected using Xcalibur 3.0 software (Thermo Fisher Scientific, MA, USA) from a single full-scan mass spectrum in Orbitrap (350–1550 m/z, 120,000 resolution) followed by a 3 s data-dependent MS/MS scan in an Ion Routing Multipole at 38% normalized collision energy (HCD).

### Data analysis

Protein identification and quantification were performed using Proteome Discoverer 2.1 software (Thermo Fisher Scientific, MA, USA) with the SEQUEST HT algorithm. Mass spectra data were extracted from raw data and searched against the SwissProt reviewed mouse protein database (released on 20161208, 16839 protein items). Precursor Mass Tolerance and Fragment Mass Tolerance were set at 20 ppm and 0.02 Da, respectively, and a maximum of two missed cleavages was allowed. Total Intensity Threshold and Minimum Peak Count were set at 20,000 and 200, respectively. Carbamidomethylation (on C) and TMT 6plex (on K and peptide N terminal) were set as static modifications, and oxidation (on M), phosphate (on S, T, Y) and deamination (on N) were set as dynamic modifications. Protein identification was considered valid if at least one peptide was statistically significant (with a false discovery rate (FDR) of 1%). Default values were used for all other parameters.

TMT 6plex was chosen as the quantification method. Proteins were quantified based on the unique peptide ratio. Search results were read using Proteome Discoverer 2.1 with high peptide confidence filters. Proteins were filtered with scores >10 and unique peptide ratios >1. The differential expression threshold was defined > 1.2-fold change or <0.85-fold change.

The mass spectrometry proteomics data have been deposited to the ProteomeXchange Consortium^48^ via the PRIDE partner repository with the data set identifier PXD006448.

### Bioinformatic analysis

For functional annotation, the differentially expressed peptides (DEPs) were subjected to functional analysis using DAVID bioinformatics resources (https://david.ncifcrf.gov). GO terms for biological processes and cellular components were obtained using default statistical parameters (threshold: count 2, ease 0.1).

Protein–protein interaction networks of DEPs were constructed with STRING App in Cytoscape 3.4. “Experimental” and “Database” evidence was set at medium (0.4) confidence level.

### Animal experimentation

Eight-week-old female FVB/N mice (Charles River Laboratories) were either ovariectomized (OVX) or sham-operated. Mice received subcutaneous implants of osmotic pumps (Alzet Model 2006, Durect Corp., Cupertino, CA, USA) delivering either EPZ5676 in 50% DMSO, 50% water at rate of 1.6 mg/d (OVX + EPZ5676) or a vehicle (sham and OVX) at day 7 after operation. The dose of EPZ5676 was based on a previous report that a similar dose inhibited tumor growth in Nude rats bearing MV4-11 xenografts^49^. Osmotic pumps were replaced with Alzet Model 2002 after six weeks and treatment was continued for two weeks. After 8 weeks of treatment, animals were sacrificed. EPZ5676 treatment was continued for 8 weeks because bone formation rate and bone volume remain stable during the period between 8 and 10 weeks post-OVX^50^, thus reducing the experimental error caused by individual differences between control and EPZ5676 treatment groups. Left hind limb from treated and control animals were collected for Histomorphometric and immunohistochemistry analysis. The right hind limb was scanned with a Scanco µCT-100 (Scanco Medical, Bruttisellen, Switzerland). All animal feeding and surgical procedures were approved by the Center for Experimental Animal Research of Institute of Basic Medical Sciences, Chinese Academy of Medical Sciences.

### Micro-CT scanning of bone

All soft tissue was removed from the femur and tibia prior to fixation for 24 h with 4% paraformaldehyde. Micro-CT (µCT) images were scanned with the Scanco µCT-100 using the following parameters: resolution 10 µm, X-ray tube potential set 70 kV, intensity 200 µA, and integration time 200 ms. The Scanco evaluation program V6.5-3 (Scanco Medical, Bruttisellen, Switzerland) was used to generate three-dimensional reconstructions from two dimensional images.

### Histomorphometric and immunohistochemistry analysis

For static histomorphometric analysis, femurs were fixed in 4% paraformaldehyde for 48 h and decalcification was performed with 10% EDTA for 14 days. The samples were then embedded in paraffin. Longitudinally oriented sections (5-μm–thick) of femur, including the metaphysis and diaphysis, were dewaxed, rehydrated with gradient ethanol, and then stained for TRAP (Sigma-Aldrich). OC surface/bone surface (Oc.S/BS, %) and OC number/bone surface (N.Oc/BS, N/mm) were measured.

For immunohistochemistry analysis, bone sections were dewaxed, rehydrated with gradient ethanol, and then incubated with a primary detection antibody (OPN, sc-21742) overnight at 4 °C. Immunoreactivity was detected with Horseradish peroxidase–streptavidin detection system (Zhongshanjinqiao, Beijing, China). Isotype-matched negative control antibodies were used as negative controls.

### Statistical analysis

Unless indicated, all statistical analysis were performed on data generated from triplicate experiments. Results are expressed as the mean ± standard deviation. For most experiments, unless indicated, statistical significance of differences was evaluated by Student’s *t-*test (two-tailed, unpaired). *P* < 0.05 was considered to indicate statistical significance.

## supplementary material

Supplementary Figures

Supplementary Table 3

Supplementary Table 4

Supplementary Table 1

Supplementary Table 2
