# Supplementary material for: The histone methyltransferase DOT1L inhibits osteoclastogenesis and protects against osteoporosis
Source: Cell Death Dis. 2018 Jan 18;9(2):33. doi: 10.1038/s41419-017-0040-5 (PMC5833786; doi:10.1038/s41419-017-0040-5)
Supplement: Supplementary file 1 — Supplementary Figures [file 41419_2017_40_MOESM1_ESM.docx]

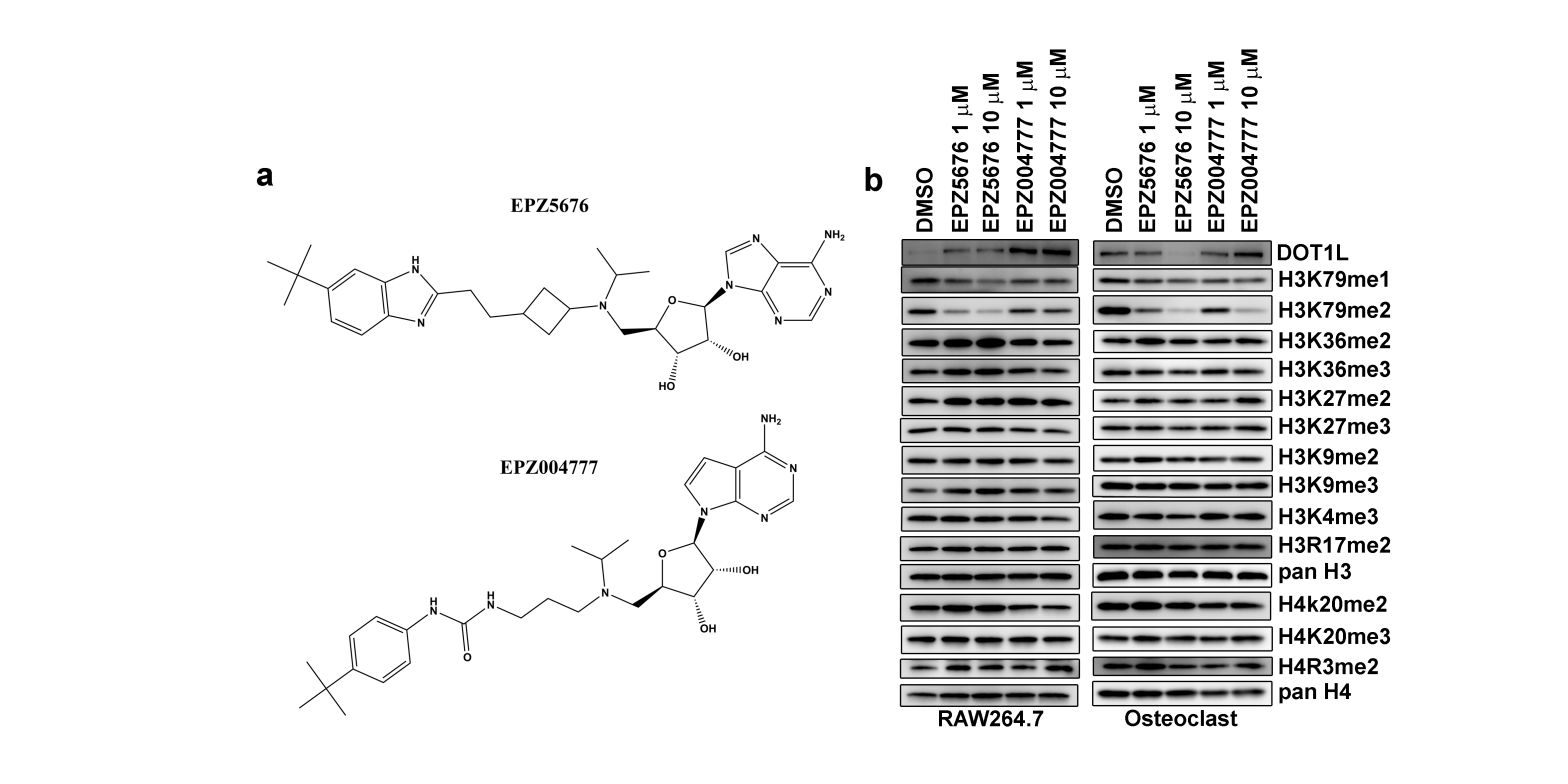


**Supplementary Figure S1. Inhibition of H3K79 methylation by DOT1L inhibitors without disturbance of other indicated histone methylation levels. a, The chemical structure of DOT1L inhibitors EPZ5676 and EPZ004777. b,** Immunoblot analysis of histones from RAW264.7 cells or osteoclasts treated for 60 h with either DOT1L inhibitors (EPZ5676 and EPZ004777) or vehicle control and probed with a panel of methyl-lysine and methyl-arginine specific antibodies. Total H3 and H4 antibodies were used as the loading controls.

**
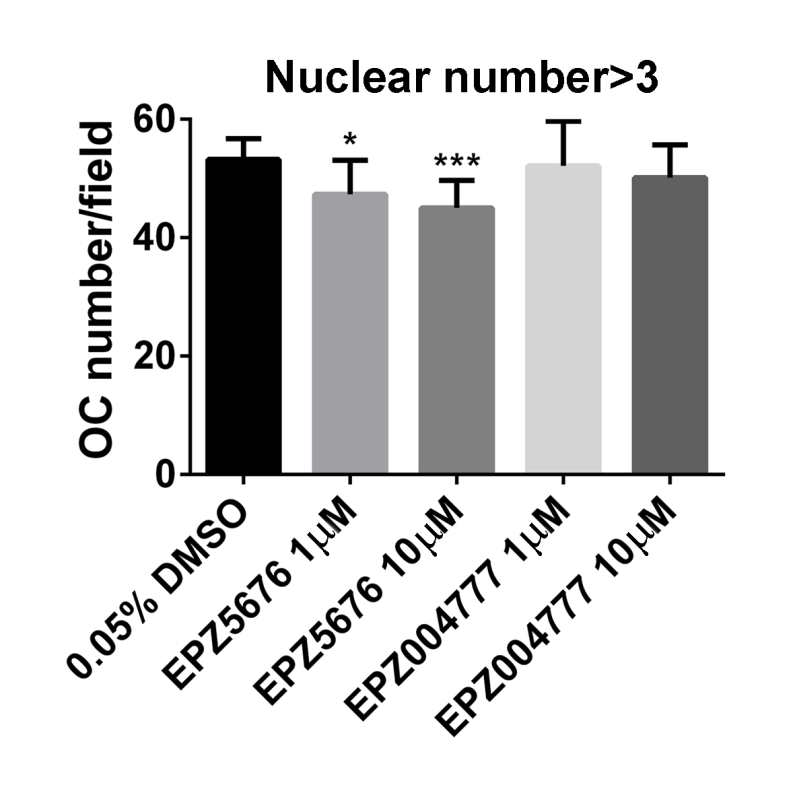
**

**Supplementary Figure S2. The number of osteoclasts with nuclear number greater than 3.** RAW264.7 cells pretreated with DMSO or the indicated concentrations of DOT1L inhibitors (EPZ5676 and EPZ004777) were stimulated for 60 h with RANKL. **P*<0.05, ***P*<0.01, ****P*<0.001, *****P*<0.0001, two-tailed, unpaired *t*-test, compared with the DMSO treatment.


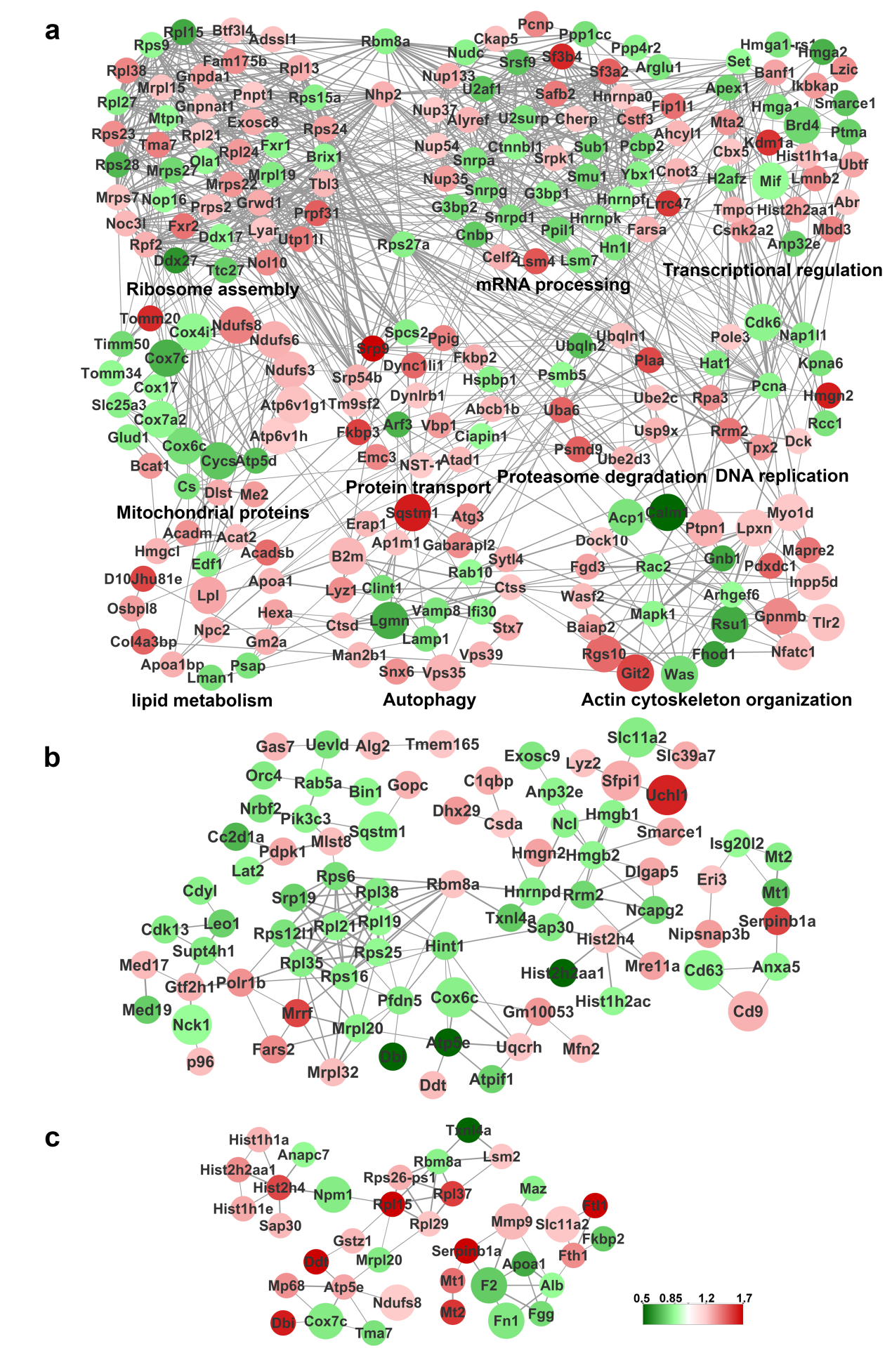


**Supplementary Figure S3. Interaction network constructed with DEPs in three stages of osteoclast formation.** DEPs were identified via proteomic approaches based on TMT-labeling in three stages of osteoclast differentiation induced by DOT1L inhibitor treatment for the indicated times; 40-h pre-osteoclasts, 60-h pre-osteoclasts, and 60-h osteoclasts. DEPs were submitted to the STRING online protein-protein-interaction system (<http://string-db.org/>). Proteins in the networks are shown as nodes. The width of the lines connecting the proteins indicates the connection score obtained from the database. Up- or downregulation is indicated by the color of nodes (upregulated in red and downregulated in green) Proteins previously reported to be associated with osteoclast differentiation are indicated in a large circle. a–c, Network of DEPs identified in 40-h pre-osteoclasts, 60-h pre-osteoclasts, and 60-h osteoclasts. Function groups are classified based on UniProt function and published reports.


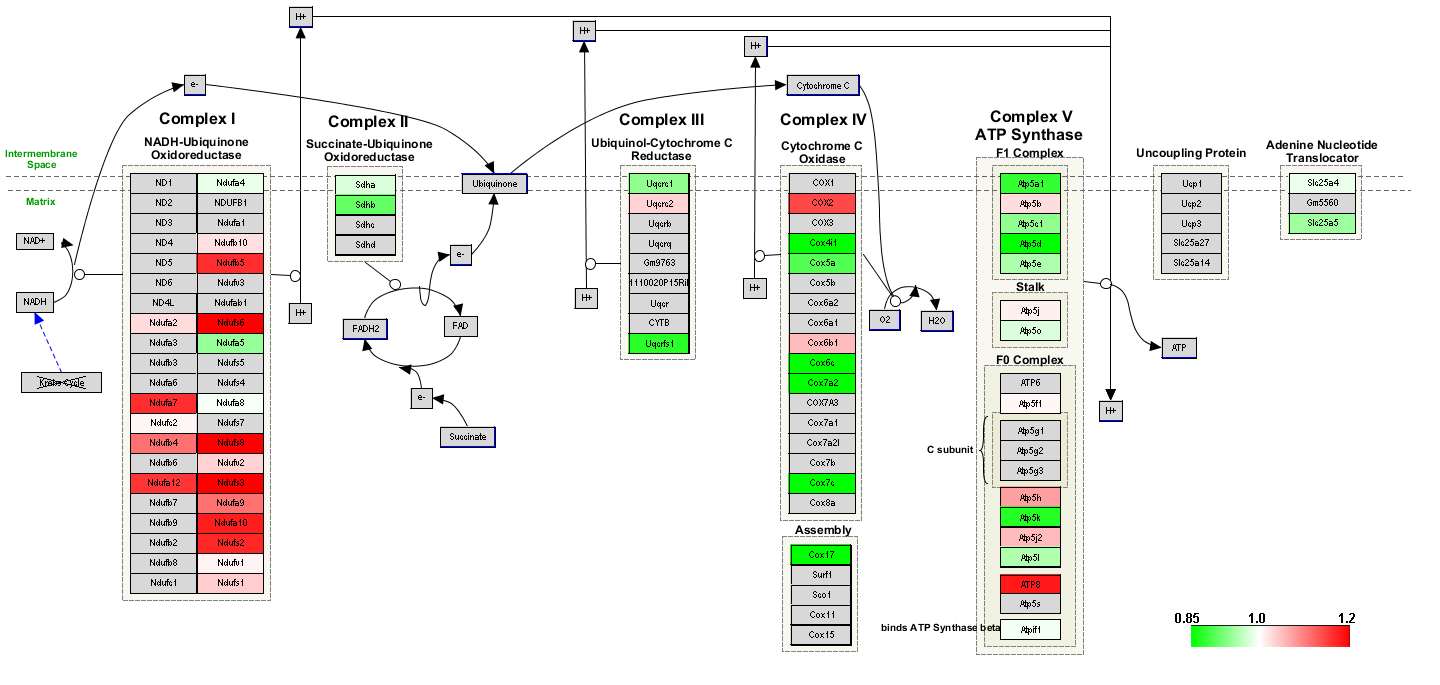


**Supplementary Figure S4. DEPs identified in the electron transport chain.** All identified DEPs in 40-h pre-osteoclasts were mapped to the electron transport chain using PathVisio. Proteins are represented by boxes labeled with the protein name. Relative protein expression levels are indicated by different colors. Proteins in gray were not identified in this study


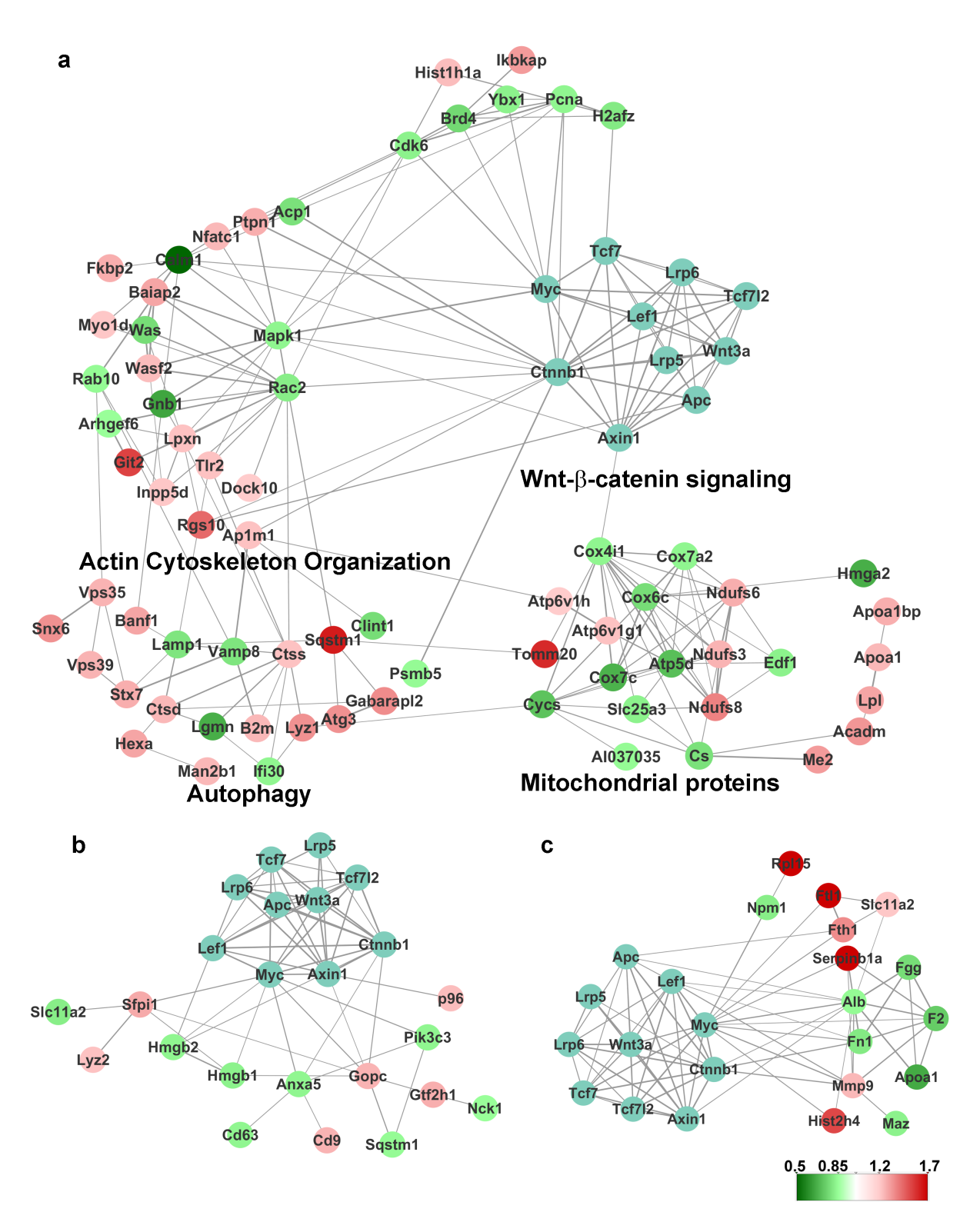


**Supplementary Figure S5. Interaction network between Wnt pathway proteins and DEPs in three stages of osteoclast formation.** The DEPs shown in Figure 6a–c were extracted and reanalyzed with Wnt pathway proteins. Protein accession numbers were submitted to the STRING online protein-protein-interaction system (<http://string-db.org/>). Proteins in the networks are shown as nodes. The width of the lines connecting the proteins indicates the connection score obtained from the database. Up- or downregulation is indicated by the color of nodes (upregulated in red and downregulated in green). Proteins in the blue node were not detected in the proteomics data. a–c, Network of the relationships between Wnt pathway proteins and DEPs identified in 40-h pre-osteoclasts, 60-h pre-osteoclasts, and 60-h osteoclasts. Function groups are classified based on UniProt function and published reports.
