# Supplementary material for: The histone methyltransferase DOT1L inhibits osteoclastogenesis and protects against osteoporosis
Source: Cell Death Dis. 2018 Jan 18;9(2):33. doi: 10.1038/s41419-017-0040-5 (PMC5833786; doi:10.1038/s41419-017-0040-5)
Supplement: Supplementary file 2 — Supplementary Table 3 [file 41419_2017_40_MOESM2_ESM.docx]

**Supplementary Table 3 the DEPs previous reports associated with osteoclast differentiation or resorption.**

| **Accession** | **Description** | **Score** | **Unique Peptides** | **EPZ5676/DMSO** | **PSMs** | **Coverage** | **Reference** |
| --- | --- | --- | --- | --- | --- | --- | --- |
| **40h pre-osteoclast Autophagy** | |  |  |  |  |  |  |
| Q64337 | Sequestosome-1; **Sqstm1** | 44.32 | 8 | 1.56 | 13 | 28.96 | (1) |
| Q9EQH3 | Vacuolar protein sorting-associated protein 35; **Vps35** | 61.19 | 11 | 1.25 | 25 | 18.22 | (2) |
| P01887 | Beta-2-microglobulin; **B2m** | 17.02 | 3 | 1.24 | 6 | 39.50 | (3) |
| O89017 | Legumain; **Lgmn** | 30.78 | 4 | 0.66 | 9 | 10.57 | (4) |
| **40h pre-osteoclast Actin cytoskeleton organization** | |  |  |  |  |  |  |
| Q05144 | Ras-related C3 botulinum toxin substrate 2; **Rac2** | 58.57 | 5 | 0.82 | 18 | 40.10 | (5, 6) |
| P63085 | Mitogen-activated protein kinase 1; **Mapk1** | 22.99 | 5 | 0.83 | 8 | 18.16 | (7, 8) |
| P62204 | Calmodulin; **Calm1** | 117.05 | 6 | 0.49 | 36 | 61.74 | (9) |
| Q01730 | Ras suppressor protein 1; **Rsu1** | 22.24 | 6 | 0.65 | 7 | 28.52 | (10) |
| P70315 | Wiskott-Aldrich syndrome protein homolog; **Was** | 22.52 | 5 | 0.77 | 7 | 12.50 | (11, 12) |
| P35821 | Tyrosine-protein phosphatase non-receptor type 1; **Ptpn1** | 16.95 | 2 | 1.26 | 5 | 6.02 | (13) |
| Q9JLQ2 | ARF GTPase-activating protein GIT2; **Git2** | 22.99 | 2 | 1.48 | 6 | 5.93 | (14) |
| Q9CQE5 | Regulator of G-protein signaling 10; **Rgs10** | 28.83 | 4 | 1.40 | 8 | 33.15 | (15, 16) |
| Q9D358 | Low molecular weight phosphotyrosine protein phosphatase; **Acp1** | 35.32 | 4 | 0.78 | 10 | 39.24 | (17) |
| Q99N69 | Leupaxin; **Lpxn** | 55.30 | 10 | 1.22 | 21 | 35.49 | (18, 19) |
| Q5SYD0 | Unconventional myosin-Id; **Myo1d** | 13.14 | 6 | 1.21 | 8 | 8.65 | (20) |
| Q9ES52 | Phosphatidylinositol 3,4,5-trisphosphate 5-phosphatase 1; **Inpp5d** | 57.11 | 17 | 1.21 | 21 | 17.80 | (21, 22) |
| Q99P91 | Transmembrane glycoprotein NMB; **Gpnmb** | 38.06 | 4 | 1.34 | 11 | 8.54 | (23, 24) |
| Q9QUN7 | Toll-like receptor 2; **Tlr2** | 13.07 | 5 | 1.22 | 5 | 7.65 | (25, 26) |
| O88942 | Nuclear factor of activated T-cells, cytoplasmic 1; **Nfatc1** | 27.56 | 8 | 1.24 | 11 | 18.55 | (27, 28) |
| **40h pre-osteoclast Mitochondrial proteins** | |  |  |  |  |  |  |
| Q9CR51 | V-type proton ATPase subunit G 1; **Atp6v1g1** | 33.40 | 3 | 1.22 | 8 | 28.81 | (29) |
| Q8BVE3 | V-type proton ATPase subunit H; **Atp6v1h** | 34.03 | 8 | 1.20 | 11 | 19.05 | (30, 31) |
| P62897 | Cytochrome c, somatic; **Cycs** | 99.76 | 15 | 0.71 | 29 | 69.52 | (32) |
| P19783 | Cytochrome c oxidase subunit 4 isoform 1, mitochondrial; **Cox4i1** | 67.03 | 7 | 0.83 | 23 | 29.59 | (33-35) |
| Q9CPQ1 | Cytochrome c oxidase subunit 6C; **Cox6c** | 16.01 | 4 | 0.77 | 7 | 31.58 | (33-35) |
| P48771 | Cytochrome c oxidase subunit 7A2, mitochondrial; **Cox7a2** | 16.03 | 2 | 0.83 | 5 | 27.71 | (33-35) |
| P17665 | Cytochrome c oxidase subunit 7C, mitochondrial; **Cox7c** | 17.33 | 2 | 0.67 | 5 | 17.46 | (33-35) |
| Q8K3J1 | NADH dehydrogenase (ubiquinone) iron-sulfur protein 8, mitochondrial; **Ndufs8** | 13.58 | 3 | 1.35 | 4 | 17.92 | (36, 37) |
| P52503 | NADH dehydrogenase (ubiquinone) iron-sulfur protein 6, mitochondrial; **Ndufs6** | 29.23 | 4 | 1.26 | 9 | 41.38 | (36, 37) |
| Q9DCT2 | NADH dehydrogenase (ubiquinone) iron-sulfur protein 3, mitochondrial; **Ndufs3** | 27.30 | 7 | 1.25 | 10 | 32.32 | (36, 37) |
| **40h pre-osteoclast Lipid metabolism** | |  |  |  |  |  |  |
| P11152 | Lipoprotein lipase; **Lpl** | 20.10 | 6 | 1.28 | 6 | 20.04 | (38) |
| **40h pre-osteoclast DNA replication** | |  |  |  |  |  |  |
| Q64261 | Cyclin-dependent kinase 6; **Cdk6** | 29.94 | 6 | 0.82 | 11 | 29.14 | (39) |
| **40h pre-osteoclast Transcriptional factor** | |  |  |  |  |  |  |
| P34884 | Macrophage migration inhibitory factor; **Mif** | 39.51 | 5 | 0.85 | 20 | 52.17 | (40-42) |
| Q9ESU6 | Bromodomain-containing protein 4; **Brd4** | 14.53 | 4 | 0.76 | 4 | 4.43 | (43-45) |
| **60h pre-osteoclast** | |  |  |  |  |  |  |
| P40240 | CD9 antigen; **Cd9** | 224.88 | 5 | 1.25 | 49 | 21.68 | (46-48) |
| P41731 | CD63 antigen; **Cd63** | 28.69 | 3 | 0.83 | 9 | 8.82 | (48) |
| P17433 | Transcription factor PU.1; **Spi1** | 33.08 | 4 | 1.27 | 10 | 11.40 | (49) |
| Q9R0P9 | Ubiquitin carboxyl-terminal hydrolase isozyme L1; **Uchl1** | 19.43 | 5 | 1.55 | 7 | 19.28 | (50) |
| P49282 | Natural resistance-associated macrophage protein 2; **Slc11a2** | 14.11 | 2 | 0.81 | 3 | 6.87 | (51) |
| Q99M51 | Cytoplasmic protein NCK1; **Nck1** | 40.74 | 11 | 0.85 | 16 | 41.64 | (52) |
| Q9CPQ1 | Cytochrome c oxidase subunit 6C; **Cox6c** | 32.30 | 6 | 0.82 | 13 | 38.16 | (33-35) |
| Q64337 | Sequestosome-1; **Sqstm1** | 64.54 | 8 | 0.84 | 20 | 29.19 | (1) |
| **60h osteoclast** | |  |  |  |  |  |  |
| P11276 | Fibronectin; **Fn1** | 22.81 | 7 | 0.80 | 8 | 2.91 | (53, 54) |
| P19221 | Prothrombin; **F2** | 23.71 | 5 | 0.73 | 7 | 8.41 | (53, 55) |
| P41245 | Matrix metalloproteinase-9; **Mmp9** | 35.31 | 9 | 1.24 | 13 | 15.07 | (56) |
| Q61937 | Nucleophosmin; **Npm1** | 1016.68 | 22 | 0.81 | 291 | 54.11 | (57, 58) |
| P49282 | Natural resistance-associated macrophage protein 2; **Slc11a2** | 14.11 | 2 | 1.21 | 3 | 6.87 | (51) |
| Q8K3J1 | NADH dehydrogenase (ubiquinone] iron-sulfur protein 8; **Ndufs8** | 78.96 | 8 | 1.20 | 26 | 36.32 | (36, 37) |
| P17665 | Cytochrome c oxidase subunit 7C; **Cox7c** | 40.72 | 4 | 0.80 | 13 | 41.27 | (33-35) |

**References**

1. McManus S & Roux S (2012) The adaptor protein p62/SQSTM1 in osteoclast signaling pathways. *Journal of molecular signaling* 7(1):1.

2. Xia W-F*, et al.* (2013) Vps35 loss promotes hyperresorptive osteoclastogenesis and osteoporosis via sustained RANKL signaling. *The Journal of Cell Biology* 200(6):821-837.

3. Menaa C, Esser E, & Sprague SM (2008) Beta2-microglobulin stimulates osteoclast formation. *Kidney international* 73(11):1275-1281.

4. Choi SJ*, et al.* (1999) Identification of human asparaginyl endopeptidase (legumain) as an inhibitor of osteoclast formation and bone resorption. *The Journal of biological chemistry* 274(39):27747-27753.

5. Croke M*, et al.* (2011) Rac deletion in osteoclasts causes severe osteopetrosis. *Journal of cell science* 124(Pt 22):3811-3821.

6. Itokowa T*, et al.* (2011) Osteoclasts lacking Rac2 have defective chemotaxis and resorptive activity. *Calcified tissue international* 88(1):75-86.

7. He Y*, et al.* (2011) Erk1 positively regulates osteoclast differentiation and bone resorptive activity. *PLoS One* 6(9):e24780.

8. Miyazaki T*, et al.* (2000) Reciprocal role of ERK and NF-κB pathways in survival and activation of osteoclasts. *The Journal of cell biology* 148(2):333-342.

9. Masuyama R*, et al.* (2012) Calcium/calmodulin-signaling supports TRPV4 activation in osteoclasts and regulates bone mass. *Journal of Bone and Mineral Research* 27(8):1708-1721.

10. Deng FY*, et al.* (2008) Proteomic analysis of circulating monocytes in Chinese premenopausal females with extremely discordant bone mineral density. *Proteomics* 8(20):4259-4272.

11. Calle Y*, et al.* (2004) WASp deficiency in mice results in failure to form osteoclast sealing zones and defects in bone resorption. *Blood* 103(9):3552-3561.

12. Chellaiah MA, Kuppuswamy D, Lasky L, & Linder S (2007) Phosphorylation of a Wiscott-Aldrich syndrome protein-associated signal complex is critical in osteoclast bone resorption. *The Journal of biological chemistry* 282(13):10104-10116.

13. Zee T, Settembre C, Levine RL, & Karsenty G (2012) T-Cell Protein Tyrosine Phosphatase Regulates Bone Resorption and Whole-Body Insulin Sensitivity through Its Expression in Osteoblasts. *Molecular and cellular biology* 32(6):1080-1088.

14. Heckel T*, et al.* (2009) Src-dependent repression of ARF6 is required to maintain podosome-rich sealing zones in bone-digesting osteoclasts. *Proceedings of the National Academy of Sciences of the United States of America* 106(5):1451-1456.

15. Yang S & Li YP (2007) RGS10-null mutation impairs osteoclast differentiation resulting from the loss of [Ca2+]i oscillation regulation. *Genes & development* 21(14):1803-1816.

16. Yang S, Chen W, Stashenko P, & Li YP (2007) Specificity of RGS10A as a key component in the RANKL signaling mechanism for osteoclast differentiation. *Journal of cell science* 120(Pt 19):3362-3371.

17. Alho I, Costa L, Bicho M, & Coelho C (2016) Low Molecular Weight Protein Tyrosine Phosphatase Slow Isoform Knockdown in MDA-MB-435 Cells Decreases RAW 264.7 Osteoclastic Differentiation. *Anticancer research* 36(5):2227-2232.

18. Sahu SN*, et al.* (2007) Association of leupaxin with Src in osteoclasts. *American journal of physiology. Cell physiology* 292(1):C581-590.

19. Gupta A*, et al.* (2003) Leupaxin is a critical adaptor protein in the adhesion zone of the osteoclast. *Journal of bone and mineral research : the official journal of the American Society for Bone and Mineral Research* 18(4):669-685.

20. Birnbaum MJ*, et al.* (2010) Using osteoclast differentiation as a model for gene discovery in an undergraduate cell biology laboratory. *Biochemistry and molecular biology education : a bimonthly publication of the International Union of Biochemistry and Molecular Biology* 38(6):385-392.

21. Zhou P*, et al.* (2006) SHIP1 negatively regulates proliferation of osteoclast precursors via Akt-dependent alterations in D-type cyclins and p27. *Journal of immunology (Baltimore, Md. : 1950)* 177(12):8777-8784.

22. Iyer S, Margulies BS, & Kerr WG (2013) Role of SHIP1 in bone biology. *Annals of the New York Academy of Sciences* 1280:11-14.

23. Sheng MH, Wergedal JE, Mohan S, & Lau KH (2008) Osteoactivin is a novel osteoclastic protein and plays a key role in osteoclast differentiation and activity. *FEBS letters* 582(10):1451-1458.

24. Sheng MH*, et al.* (2012) Targeted overexpression of osteoactivin in cells of osteoclastic lineage promotes osteoclastic resorption and bone loss in mice. *PLoS One* 7(4):e35280.

25. Kassem A, Lindholm C, & Lerner UH (2016) Toll-Like Receptor 2 Stimulation of Osteoblasts Mediates Staphylococcus Aureus Induced Bone Resorption and Osteoclastogenesis through Enhanced RANKL. *PLoS One* 11(6):e0156708.

26. Chen Z*, et al.* (2015) IL-1R/TLR2 through MyD88 Divergently Modulates Osteoclastogenesis through Regulation of Nuclear Factor of Activated T Cells c1 (NFATc1) and B Lymphocyte-induced Maturation Protein-1 (Blimp1). *The Journal of biological chemistry* 290(50):30163-30174.

27. Takayanagi H*, et al.* (2002) Induction and Activation of the Transcription Factor NFATc1 (NFAT2) Integrate RANKL Signaling in Terminal Differentiation of Osteoclasts. *Developmental Cell* 3(6):889-901.

28. Kim K, Lee S-H, Kim JH, Choi Y, & Kim N (2008) NFATc1 Induces Osteoclast Fusion Via Up-Regulation of Atp6v0d2 and the Dendritic Cell-Specific Transmembrane Protein (DC-STAMP). *Molecular Endocrinology* 22(1):176-185.

29. Tan L-J*, et al.* (2015) Bivariate Genome-Wide Association Study Implicates ATP6V1G1 as a Novel Pleiotropic Locus Underlying Osteoporosis and Age at Menarche. *The Journal of Clinical Endocrinology & Metabolism* 100(11):E1457-E1466.

30. Zhang Y*, et al.* (2017) ATP6V1H Deficiency Impairs Bone Development through Activation of MMP9 and MMP13. *PLOS Genetics* 13(2):e1006481.

31. Duan X*, et al.* (2016) Deficiency of ATP6V1H Causes Bone Loss by Inhibiting Bone Resorption and Bone Formation through the TGF-β1 Pathway. *Theranostics* 6(12):2183-2195.

32. Oursler MJ, Bradley EW, Elfering SL, & Giulivi C (2005) Native, not nitrated, cytochrome c and mitochondria-derived hydrogen peroxide drive osteoclast apoptosis. *American journal of physiology. Cell physiology* 288(1):C156-168.

33. Miyazaki T, Neff L, Tanaka S, Horne WC, & Baron R (2003) Regulation of cytochrome <em>c</em> oxidase activity by c-Src in osteoclasts. *The Journal of Cell Biology* 160(5):709-718.

34. Noda K, Tani N, Nakamura Y, & Kuwahara Y (1994) Cytochrome c oxidase activity in osteoclasts appeared in an early stage of parathyroid hormone treatment. *Journal of electron microscopy* 43(3):168-172.

35. Fukushima O, Bekker PJ, & Gay CV (1991) Characterization of the functional stages of osteoclasts by enzyme histochemistry and electron microscopy. *The Anatomical record* 231(3):298-315.

36. Jin Z, Wei W, Yang M, Du Y, & Wan Y (2014) Mitochondrial complex I activity suppresses inflammation and enhances bone resorption by shifting macrophage-osteoclast polarization. *Cell metabolism* 20(3):483-498.

37. Huang Stanley C-C & Pearce Edward J (2014) For Macrophages, Ndufs Is Enough. *Immunity* 41(3):351-353.

38. Dragojevic J*, et al.* (2013) Triglyceride metabolism in bone tissue is associated with osteoblast and osteoclast differentiation: a gene expression study. *J Bone Miner Metab* 31(5):512-519.

39. Ogasawara T*, et al.* (2004) Osteoclast differentiation by RANKL requires NF-kappaB-mediated downregulation of cyclin-dependent kinase 6 (Cdk6). *Journal of bone and mineral research : the official journal of the American Society for Bone and Mineral Research* 19(7):1128-1136.

40. Madeira MF*, et al.* (2012) MIF induces osteoclast differentiation and contributes to progression of periodontal disease in mice. *Microbes and infection* 14(2):198-206.

41. Gu R*, et al.* (2015) Macrophage migration inhibitory factor is essential for osteoclastogenic mechanisms in vitro and in vivo mouse model of arthritis. *Cytokine* 72(2):135-145.

42. Movila A*, et al.* (2016) Macrophage Migration Inhibitory Factor (MIF) Supports Homing of Osteoclast Precursors to Peripheral Osteolytic Lesions. *Journal of bone and mineral research : the official journal of the American Society for Bone and Mineral Research* 31(9):1688-1700.

43. Baud'huin M*, et al.* (2017) Inhibition of BET proteins and epigenetic signaling as a potential treatment for osteoporosis. *Bone* 94:10-21.

44. Deepak V*, et al.* (2016) In silico design and bioevaluation of selective benzotriazepine BRD4 inhibitors with potent antiosteoclastogenic activity. *Chemical biology & drug design*.

45. Meng S*, et al.* (2014) BET Inhibitor JQ1 Blocks Inflammation and Bone Destruction. *Journal of dental research* 93(7):657-662.

46. Ishii M*, et al.* (2006) RANKL-induced expression of tetraspanin CD9 in lipid raft membrane microdomain is essential for cell fusion during osteoclastogenesis. *Journal of bone and mineral research : the official journal of the American Society for Bone and Mineral Research* 21(6):965-976.

47. Yi T*, et al.* (2006) Tetraspanin CD9 regulates osteoclastogenesis via regulation of p44/42 MAPK activity. *Biochemical and biophysical research communications* 347(1):178-184.

48. Parthasarathy V*, et al.* (2009) Distinct roles for tetraspanins CD9, CD63 and CD81 in the formation of multinucleated giant cells. *Immunology* 127(2):237-248.

49. Sharma SM*, et al.* (2007) MITF and PU. 1 recruit p38 MAPK and NFATc1 to target genes during osteoclast differentiation. *Journal of Biological Chemistry* 282(21):15921-15929.

50. Coudert AE*, et al.* (2014) Differentially expressed genes in autosomal dominant osteopetrosis type II osteoclasts reveal known and novel pathways for osteoclast biology. *Lab Invest* 94(3):275-285.

51. Xie W, Lorenz S, Dolder S, & Hofstetter W (2016) Extracellular Iron is a Modulator of the Differentiation of Osteoclast Lineage Cells. *Calcified tissue international* 98(3):275-283.

52. Aryal ACS*, et al.* (2013) Nck1 deficiency accelerates unloading-induced bone loss. *Journal of cellular physiology* 228(7):1397-1403.

53. Hu Y, Ek-Rylander B, Karlström E, Wendel M, & Andersson G (2008) Osteoclast size heterogeneity in rat long bones is associated with differences in adhesive ligand specificity. *Experimental cell research* 314(3):638-650.

54. Hegan PS, Ostertag E, Geurts AM, & Mooseker MS (2015) Myosin Id is required for planar cell polarity in ciliated tracheal and ependymal epithelial cells. *Cytoskeleton* 72(10):503-516.

55. Karlstrom E*, et al.* (2011) Localization and expression of prothrombin in rodent osteoclasts and long bones. *Calcified tissue international* 88(3):179-188.

56. Okada Y*, et al.* (1995) Localization of matrix metalloproteinase 9 (92-kilodalton gelatinase/type IV collagenase= gelatinase B) in osteoclasts: implications for bone resorption. *Laboratory investigation; a journal of technical methods and pathology* 72(3):311-322.

57. Hattori Y*, et al.* (2014) Development of a Novel Phthalimide Derivative, Preclinical Effects on High-Risk Myeloma Cells and Osteoclasts. *Blood* 124(21):5718-5718.

58. Matsushita M*, et al.* (2015) A novel phthalimide derivative, TC11, has preclinical effects on high-risk myeloma cells and osteoclasts. *PloS one* 10(1):e0116135.
