# Supplementary material for: The histone methyltransferase DOT1L inhibits osteoclastogenesis and protects against osteoporosis
Source: Cell Death Dis. 2018 Jan 18;9(2):33. doi: 10.1038/s41419-017-0040-5 (PMC5833786; doi:10.1038/s41419-017-0040-5)
Supplement: Supplementary file 3 — Supplementary Table 4 [file 41419_2017_40_MOESM3_ESM.docx]

**Supplementary Table 4 Actin cytoskeleton regulation related DEPs**

| **Accession** | **Description** | **Score** | **Unique Peptides** | **130/128** | **PSMs** | **Coverage** |
| --- | --- | --- | --- | --- | --- | --- |
| **Rac activity regulation** | | | | | | |
| Q05144 | Ras-related C3 botulinum toxin substrate 2; **Rac2** | 58.57 | 5 | 0.82 | 18 | 40.10 |
| P62874 | Guanine nucleotide-binding protein G(I)/G(S)/G(T) subunit beta-1; **Gnb1** | 57.55 | 3 | 0.64 | 18 | 30.59 |
| P62204 | Calmodulin; **Calm1** | 117.05 | 6 | 0.49 | 36 | 61.74 |
| Q01730 | Ras suppressor protein 1; **Rsu1** | 22.24 | 6 | 0.65 | 7 | 28.52 |
| Q8K4I3 | Rho guanine nucleotide exchange factor 6; **Arhgef6** | 41.34 | 8 | 0.85 | 13 | 16.47 |
| Q6P9Q4 | FH1/FH2 domain-containing protein 1; **Fhod1** | 11.88 | 2 | 0.63 | 3 | 4.01 |
| P63085 | Mitogen-activated protein kinase 1; **Mapk1** | 22.99 | 5 | 0.83 | 8 | 18.16 |
| P70315 | Wiskott-Aldrich syndrome protein homolog; **Was** | 22.52 | 5 | 0.77 | 7 | 12.50 |
| P35821 | Tyrosine-protein phosphatase non-receptor type 1; **Ptpn1** | 16.95 | 2 | 1.26 | 5 | 6.02 |
| Q9JLQ2 | ARF GTPase-activating protein GIT2; **Git2** | 22.99 | 2 | 1.48 | 6 | 5.93 |
| Q9CQE5 | Regulator of G-protein signaling 10; **Rgs10** | 28.83 | 4 | 1.40 | 8 | 33.15 |
| **Cdc42 activity regulation** | | | | | | |
| Q8BZN6 | Dedicator of cytokinesis protein 10; **Dock10** | 15.79 | 8 | 1.20 | 8 | 4.93 |
| O88842 | FYVE, RhoGEF and PH domain-containing protein 3; **Fgd3** | 11.77 | 4 | 1.29 | 5 | 9.14 |
| Q8BKX1 | Brain-specific angiogenesis inhibitor 1-associated protein 2; **Baiap2** | 13.75 | 4 | 1.28 | 6 | 11.40 |
| Q8BH43 | Wiskott-Aldrich syndrome protein family member 2; **Wasf2** | 24.33 | 5 | 1.23 | 10 | 13.48 |
| **RhoA activity regulation** | | | | | | |
| Q9D358 | Low molecular weight phosphotyrosine protein phosphatase; **Acp1** | 35.32 | 4 | 0.78 | 10 | 39.24 |
| **Actin binding** | | | | | | |
| Q99N69 | Leupaxin; **Lpxn** | 55.30 | 10 | 1.22 | 21 | 35.49 |
| Q5SYD0 | Unconventional myosin-Id; **Myo1d** | 13.14 | 6 | 1.21 | 8 | 8.65 |
| **Cell adhesion** | | | | | | |
| Q99K01 | Pyridoxal-dependent decarboxylase domain-containing protein 1; **Pdxdc1** | 16.01 | 6 | 1.41 | 6 | 12.45 |
| Q8R001 | Microtubule-associated protein RP/EB family member 2; **Mapre2** | 10.89 | 3 | 1.37 | 4 | 17.48 |
| Q9ES52 | Phosphatidylinositol 3,4,5-trisphosphate 5-phosphatase 1; **Inpp5d** | 57.11 | 17 | 1.21 | 21 | 17.80 |
| **Cell migration** | | | | | | |
| Q99P91 | Transmembrane glycoprotein NMB; **Gpnmb** | 38.06 | 4 | 1.34 | 11 | 8.54 |
| Q9QUN7 | Toll-like receptor 2; **Tlr2** | 13.07 | 5 | 1.22 | 5 | 7.65 |
| O88942 | Nuclear factor of activated T-cells, cytoplasmic 1; **Nfatc1** | 27.56 | 8 | 1.24 | 11 | 18.55 |
| **Arp2/3 complex protein** | | | | | | |
| P61161 | Actin-related protein 2; **Actr2** | 141.39 | 12 | 0.92 | 40 | 28.93 |
| Q99JY9 | Actin-related protein 3 ; **Actr3** | 219.28 | 19 | 1.07 | 59 | 59.57 |
| Q9WV32 | Actin-related protein 2/3 complex subunit 1B; **Arpc1b** | 101.02 | 13 | 0.90 | 37 | 45.43 |
| Q9JM76 | Actin-related protein 2/3 complex subunit 3; **Arpc3** | 89.56 | 15 | 0.91 | 34 | 74.16 |
| Q9CPW4 | Actin-related protein 2/3 complex subunit 5; **Arpc5** | 77.24 | 11 | 1.03 | 22 | 82.78 |
| Q9CVB6 | Actin-related protein 2/3 complex subunit 2; **Arpc2** | 62.51 | 12 | 0.92 | 25 | 35.00 |
| P59999 | Actin-related protein 2/3 complex subunit 4; **Arpc4** | 54.57 | 10 | 1.03 | 20 | 57.14 |
